# New insight into the epidemiological trends of respiratory syncytial virus infection and the underlying anti-respiratory syncytial virus mechanisms of andrographolide: integrating Global Burden of Disease database, network pharmacological analysis, and *in vitro* experiments

**DOI:** 10.1128/spectrum.02341-25

**Published:** 2025-11-13

**Authors:** Lianzhan Huang, Buliduhong Halihaman, Yiyue Han, Jieru Zhou, Changjiang Liu, Haoxue Bai, Xuansheng Ding

**Affiliations:** 1 School of Basic Medicine and Clinical Pharmacy, China Pharmaceutical University 56651, Nanjing, Jiangsu Province, China; American University of Beirut, Riad El-Solh, Beirut, Lebanon

**Keywords:** andrographolide, respiratory syncytial virus, anti-RSV mechanisms, CX3CR1, global trends and burdens, viral adsorption inhibition, ROS/TXNIP/NF-κB pathway

## Abstract

Respiratory syncytial virus (RSV) infection causes severe burdens and harms worldwide every year, lacking specific therapeutic drugs. Andrographolide is a marketed agent, whose antiviral mechanisms between RSV and the host remain unknown. In this report, we first uncover the global disease burdens caused by RSV infection via Global Burden of Disease database analysis to raise public awareness. Results showed that the overall RSV infection mortality and trends decreased globally during 1990–2021, but children and the elderly remained high-risk populations, and the disease burdens are still severe. Then, an *in vitro* experiment was performed to reveal the effects of andrographolide, a promising anti-RSV strategy, on the RSV lifecycle and key processes. Meanwhile, molecular docking, cellular heat migration assays (cellular thermal shift assay [CETSA]), and immunoblotting were integrated to screen and identify the efficacy of andrographolide on key RSV adsorption receptors. Notably, the inhibition of andrographolide on RSV viral adsorption was found to be better than ribavirin, and the corresponding key adsorption receptor was further identified as CX3CR1. Subsequently, downstream targets and signaling pathways were unveiled via network pharmacological analysis. Finally, andrographolide was validated to significantly inhibit the expression of reactive oxygen species (ROS), TNF-α, IL-6, and IL-1β after RSV infection via the regulation of the ROS/TXNIP/NF-κB pathway. Our findings highlight that the disease burdens caused by RSV infection are still grim, deserving attention. Andrographolide, a promising anti-RSV drug, was first identified as suppressing RSV adsorption to host cells by affecting CX3CR1 and inhibiting the ROS/TXNIP/NF-κB pathway, thereby reducing oxidative stress and inflammatory responses.

**IMPORTANCE:**

The respiratory syncytial virus (RSV) is the leading pathogen responsible for acute lower respiratory tract infections globally in infants, children, the elderly, and immunocompromised individuals, warranting widespread attention. Due to its complex pathogenic mechanism, there are currently no specific drugs available. Andrographolide, a marketed drug, has long been known for its efficacy against viral infections and is frequently prescribed in China for the treatment of upper respiratory tract infections; however, the precise mechanisms of its anti-RSV action remain unclear. Our study aimed to unveil the new global epidemiological trends and burdens caused by RSV to attract attention and elucidate the antiviral mechanisms of andrographolide against the RSV, presenting data that support its unique potential as a therapeutic agent capable of flexibly adapting to treat RSV infection. These findings pave the way for developing andrographolide as a new anti-RSV drug and expanding clinical therapeutic options.

## INTRODUCTION

Respiratory syncytial virus (RSV), a common RNA virus, belongs to the *Orthopneumovirus* genus within the Pneumoviridae family and the Mononegavirales order (1, 2). RSV is one of the leading pathogens responsible for respiratory tract infections and related diseases, primarily affecting infants, the elderly, and immunocompromised patients worldwide (3). Significantly, RSV-related respiratory infection has become a major public health problem worldwide. Meanwhile, acute lower respiratory tract infection (ALRTI) caused by RSV is one of the leading causes of hospitalization (4). It can be transmitted through respiratory droplets from coughing or sneezing, virus droplets entering the eyes, nose, or mouth, pollutants, and direct contact with infected individuals, with a high incidence rate and mortality, causing a heavy burden on families and society. In 2019, there were approximately 33 million reported cases of RSV-related ALRTI worldwide, of which 3.6 million required hospitalization, and 101,400 children aged 0–5 years died from RSV-related ALRTIs (5). The latest research indicates that although RSV poses a significant threat to public health, monitoring and reporting systems for RSV are still incomplete in many countries, leading to an underestimation of its true disease burdens. In addition, the public’s understanding of RSV is relatively limited, and many parents may mistake its symptoms for the common cold, thereby delaying treatment (6). In China, much research has reported the epidemiology and clinical characteristics of RSV infection (7, 8), but there is still a lack of nationwide epidemiological research on RSV infection. Hence, it is of great importance to enhance people’s awareness and comprehensive understanding of the harms and impacts of RSV infection.

Most notably, to date, there is no effective treatment specifically for RSV infection worldwide (9, 10). Antiviral therapy mainly uses broad-spectrum antiviral drugs such as ribavirin and interferon, but their efficacy in treating RSV infection is controversial (11). Accordingly, there is still a great need for effective antivirals for the treatment of RSV infection. The development of effective antiviral therapies could reduce the disease burden and improve patient outcomes, particularly in high-risk populations. In addition, common symptoms of RSV infection include a runny nose, decreased appetite, coughing, sneezing, fever, wheezing, etc. Moreover, children infected with RSV may develop short-term complications, including otitis media, pneumonia, heart failure, encephalitis, and encephalopathy caused by severe RSV infection, and long-term complications like impaired lung function, recurrent wheezing, and asthma. Adult patients typically experience complications such as acute exacerbation of chronic obstructive pulmonary disease and cardiovascular events (congestive heart failure, acute coronary syndrome, etc.) (10). These symptoms and complications also require corresponding treatment; however, some medications may not be suitable for infants, young children, and the elderly. In conclusion, the discovery and development of effective drugs for treating RSV infection is extremely important.

Andrographolide, a diterpenoid lactone chemical compound (Fig. S1), is extracted from *Andrographis paniculata* (Burm. F.) Nees (12). Andrographolide, which possesses heat-clearing, detoxifying, antibacterial, and anti-inflammatory properties, has been approved for the treatment of respiratory infections for several decades in China. In the clinic, it has various dosage forms including tablets, capsules, and drop pills. Pharmacological studies have confirmed that andrographolide has antiviral (13), antibacterial (14), anti-inflammatory (15), analgesic, antipyretic (16), and anti-tumor (17) properties, as well as a cardiovascular protective effect. In clinical practice, andrographolide and preparations containing andrographolide have been widely used for the treatment of respiratory tract infections (18). These indicate that andrographolide is an effective alternative drug for the treatment of RSV infection. However, it is still unclear how andrographolide exerts anti-RSV effects, and its anti-RSV mechanism remains unknown, hindering its further application in treating RSV infection.

In the current study, we aimed to provide new insights into the global epidemiology and disease burdens caused by RSV infection, as well as a new therapeutic drug strategy for the treatment of RSV infection. To achieve our targets, we first performed a systematic analysis of global epidemiology and disease burden caused by RSV infections based on data extracted from the Global Burden of Disease database (GBD, https://www.healthdata.org/research-analysis/gbd). Subsequently, we comprehensively explored the anti-RSV characteristics of andrographolide, a promising new therapeutic strategy for RSV infection treatment, by integrating analyses of its effects on RSV life cycle, network pharmacological analysis, and molecular biology experiments. Key findings in our study showed that the overall epidemiological trends of RSV have declined, but economically underdeveloped areas are still the hardest hit. The infants, the elderly, and those with limited immune function are the main affected populations, deserving attention. Importantly, we found that the anti-RSV efficacy of andrographolide is mainly characterized by competitive inhibition of the adsorption process between RSV and the host for the first time. Its main adsorption receptor was identified as C-X3-C motif chemokine receptor 1 (CX3CR1). In addition, we have proved that andrographolide can also inhibit the oxidative stress and inflammatory response mediated by RSV virus adsorption on the host. Taken together, these findings provide new insights into the global RSV infection epidemiological trends and burdens and a novel understanding of the mechanism of andrographolide in treating RSV infection.

## RESULTS

### Global epidemiological trends and burdens of RSV infections

**Fig 1:**
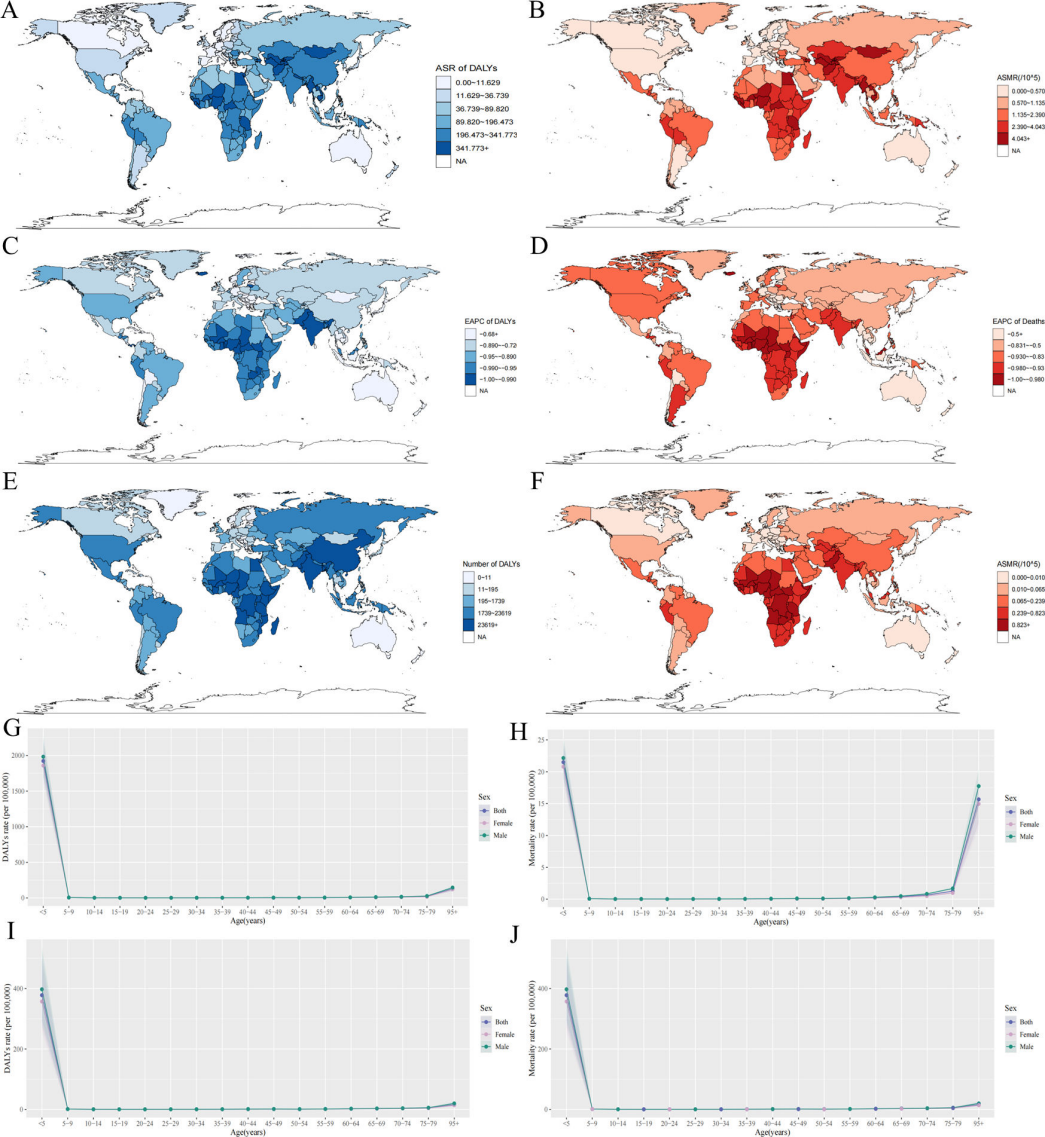
Analysis of the global epidemiological trends and burdens caused by RSV infections based on the GBD database. (**A**) ASR of DALYs in 1990 worldwide. (**B**) ASMR of DALYs in 1990 worldwide. (**C**) Estimated annual percentage change (EAPC) of DALYs during 1990–2021 worldwide. (**D**) EAPC of deaths during 1990–2021 worldwide. (**E**) ASR of DALYs in 2021 worldwide. (**F**) ASMR of DALYs in 2021 worldwide. (**G**) Change in trends of the rate of DALYs in different sex and age groups in 1990. (**H**) Change in trends of mortality rate in different sex and age groups in 1990. (**I**) Change in trends of the rate of DALYs in different sex and age groups in 2021. (**J**) Change in trends of mortality rate in different sex and age groups in 2021.

The results of the analysis showed that the number of deaths caused by RSV infection worldwide had decreased from 139,762 (95% UI: 123,666–158,111) in 1990 to 31,525 (95% UI: 23,348–41,871) in 2021 (Table S1). The disability-adjusted life years (DALYs) were decreased from 12,105,847 in 1990 (95% UI: 10,665,949–13,740,785) to 2,591,507 in 2021 (95% UI: 1,902,004–3,468,792). The related age-standardized rate (ASR) was decreased from 193.49 (95% UI: 170.61, 219.46) in 1990 to 40.82 (95% UI: 29.91, 54.59) in 2021 (Fig. 1A, C, and E) (Table S2). Age- standardized mortality rate (ASMR) decreased from 2.31 (95% UI: 2.05, 2.6) in 1990 to 0.49 (95% UI: 0.36, 0.65) in 2021 (Fig. 1B, D, and F) (Table S1). Compared with 1990, the number of deaths, mortality rates, ASMR, DALYs, and ASR caused by RSV infection in different SDI regions has significantly decreased in 2021 (Tables S1 and S2). However, we found that the death toll in the low SDI areas was about 52 times higher than the high SDI areas, indicating that the RSV burden is still severe in lower economic level areas and cannot be ignored (Table S1). Further analysis revealed that in 1990 and 2021, the top three GBD regions with the highest ASMR, age-standardized DALYs, and ASR caused by RSV were mainly concentrated in economically underdeveloped areas such as Central Asia, West Sub-Saharan Africa, and East Sub-Saharan Africa (Tables S1 and S2).

**Fig 2:**
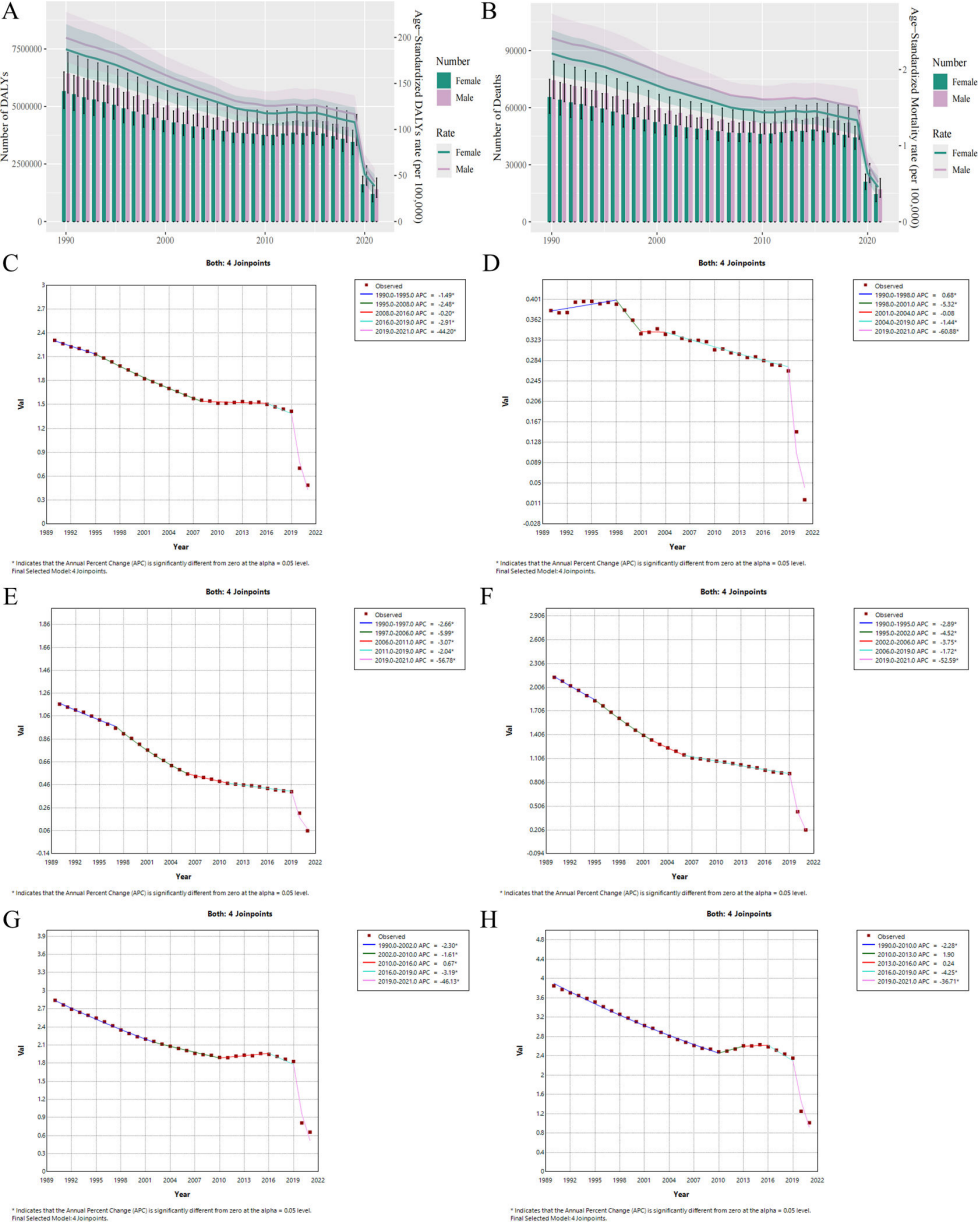
Comparative analysis of the number of DALYs and deaths associated with RSV-related LRI burden and trends of ASR changes in different genders and age groups, as well as the trends of ASMR changes in the global and different SDI regions, from 1990 to 2021. (**A**) Changed trends in the DALYs and DALY-related ASR during 1990–2021. (**B**) Changed trends in the number of deaths and ASMR during 1990–2021. (**C**) The global changed trends of ASMRs caused by RSV infections during 1990–2021. (**D**) The global changed trends of high SDI district ASMRs caused by RSV infections during 1990–2021. (**E**) The global changed trends of medium-high SDI district ASMRs caused by RSV infections during 1990–2021. (**F**) The global changed trends of medium SDI district ASMRs caused by RSV infections during 1990–2021. (**G**) The global changed trends of medium-low SDI district ASMRs caused by RSV infections during 1990–2021. (**H**) The global changed trends of low SDI district ASMRs caused by RSV infections during 1990–2021.

The analysis of the number of DALYs and deaths associated with RSV-related lower respiratory infection (LRI) burden, as well as the trend of ASR changes in different genders and age groups, showed that in 1990 and 2021, the mortality rate and DALY rate among children under 5 years and adults aged over 80 years were relatively high. Although the DALY rate in 2021 has significantly decreased compared with 1990, noticeably, the mortality rate among children under 5 years in 2021 is still relatively severe (Fig. 1G through J). The death toll and ASMR results of different genders indicated that the burden of RSV-related LRI is more severe in males than in females, and there is a similar trend in DALYs and age-standardized DALYs (Fig. 2A and B). Meanwhile, the epidemiological characteristics of RSV-related lower respiratory tract infections worldwide and in different SDI regions were also analyzed based on Joinpoint regression analysis. Between 2019 and 2021, the overall ASMR showed a downward trend globally and in five different SDI regions, with the most significant decline observed. Analysis of average annual percent change (AAPC) in different SDI regions revealed that the decline trend was most significant in the medium to high SDI and medium SDI regions, with AAPC values of −8.47 and −7.27, respectively (*P* < 0.05), while the decline trend was slowest in the low SDI region with AAPC = −4.55 (*P* < 0.05) (Fig. 2C through G) (Table S3).

In addition, we further fitted an age period cohort model and used endogenous factor analysis to quantitatively analyze the age effect, period effect, and cohort effect of RSV mortality trends from 1992 to 2021. The age effect results indicate that the risk of dying from RSV infection decreases first and then increases with age. Among them, infants and young children aged 0–4 have the highest risk of death, but after the age of 50, the mortality rate caused by RSV infection gradually increases with age. The period effect results indicate that the mortality rate and DALY rate gradually decreased with the increase of the birth cohort from 1992 to 2001. Meanwhile, the cohort effect results indicate that from 1992 to 2021, the mortality rate and DALY rate caused by RSV infection showed an overall decreasing trend (Fig. S2A through G). Moreover, predicting RSV-related LRI burden in the next 15 years (2019–2034) based on the Auto Regressive Integrated Moving Average (ARIMA) model, the trend of ASMR caused by RSV infection will continue to decrease in the next 15 years and may decrease from 1.42 in 2019 to 1.01 in 2034 (Fig. S2H and I).

Taken together, the overall RSV mortality and DALY rates have decreased globally from 1990 to 2021; however, children and the elderly remain high-risk populations. Especially in the low SDI regions with relatively backward economic and cultural levels, their burdens of RSV disease are still relatively severe, deserving attention.

### RSV proliferation and cytotoxicity assay

**Fig 3:**
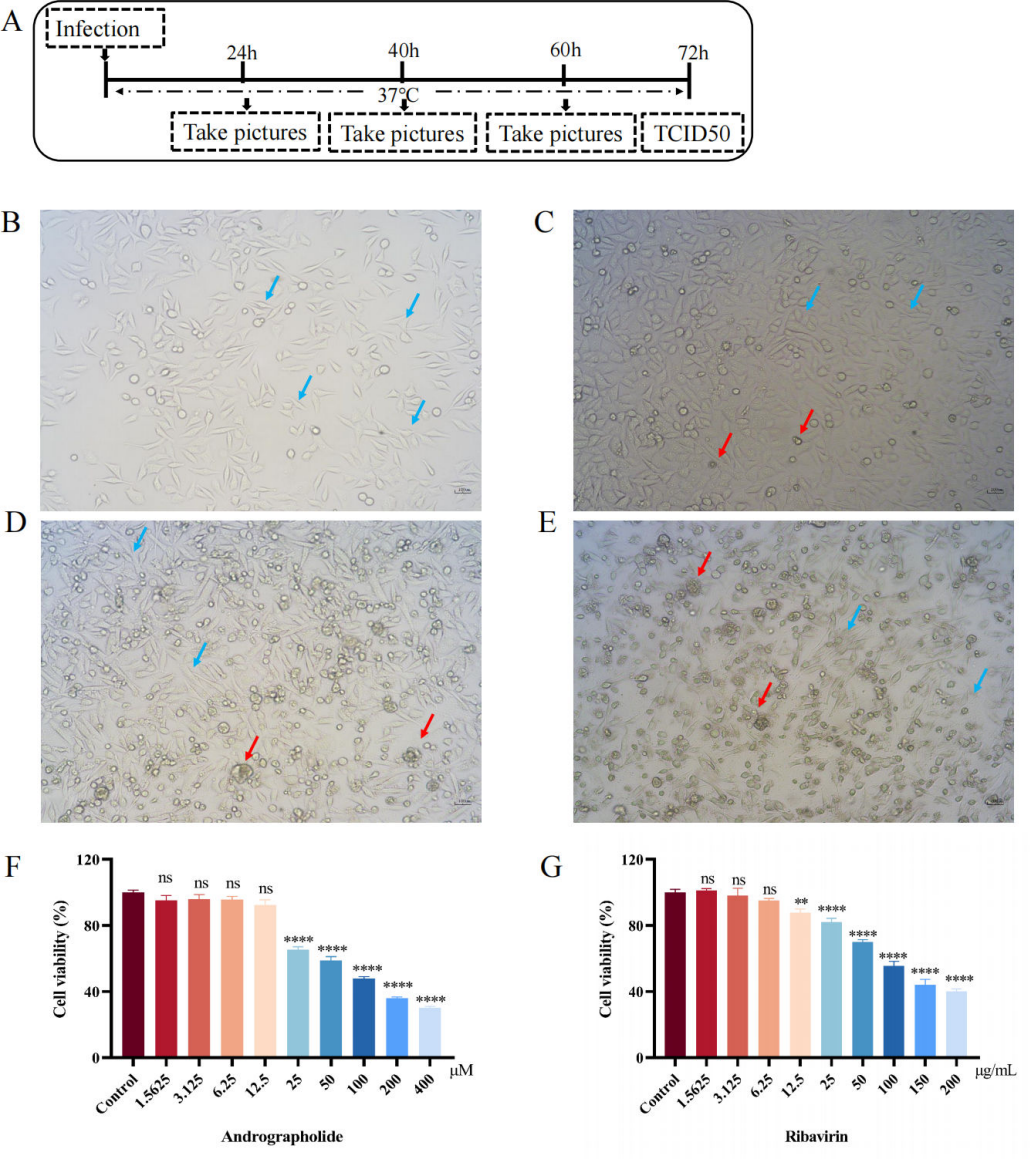
The TCID50 detection of RSV proliferation, and the cytotoxic effects of andrographolide on HEp-2 cells. (**A**) Time schedule for picture or sample collection for RS virus infection experiments and TCID50 detection. (**B**) HEp-2 cells in normal morphology. (**C**) Morphology of HEp-2 cells after RSV infection for 24 h. (**D**) Morphology of HEp-2 cells after RSV infection for 40 h. (**E**) Morphology of HEp-2 cells after 60 h of RSV infection. (**F**) The cytotoxic effects of serial concentrations of andrographolide (1.5625, 3.125, 6.25, 12.5, 25, 50, 100, 200, and 400 μΜ) on HEp-2 cells detected by MTT assays. (**G**) The cytotoxic effects of serial concentrations of ribavirin (1.5625, 3.125, 6.25, 12.5, 25, 50, 100, and 200 µg/mL) on HEp-2 cells detected by MTT assays. One-way ANOVA with Dunnett was used for statistical analysis. ****P < 0.0001; **P < 0.01; and ns, *P* > 0.05 versus the control group.

Figure 3A illustrates the RSV proliferation and TCID50 detection scheme. Before investigating the cytotoxic effects of andrographolide on HEp-2 cells, we first determined the TCID50 value of RSV, which was found to be 10^-5.52^ (Table S4). Meanwhile, the process of RSV lesions caused by HEp-2 infection was recorded during the study (Fig. 3B through E). Then, the cell viability effects of andrographolide on HEp-2 cells were detected by MTT assays. The results showed that concentrations of andrographolide lower than 25 μΜ have no significant cytotoxic inhibition on HEp-2 cells (Fig. 3F). While the non-cytotoxic concentrations of ribavirin are lower than 6.25 µg/mL (Fig. 3G). Therefore, the non-cytotoxic concentrations of andrographolide (3.125, 6.25, and 12.5 μΜ) and ribavirin (6.25 µg/mL) were selected for the following formal study.

### Determination of andrographolide administration time and its effects on RSV cycle-related protein

**Fig 4:**
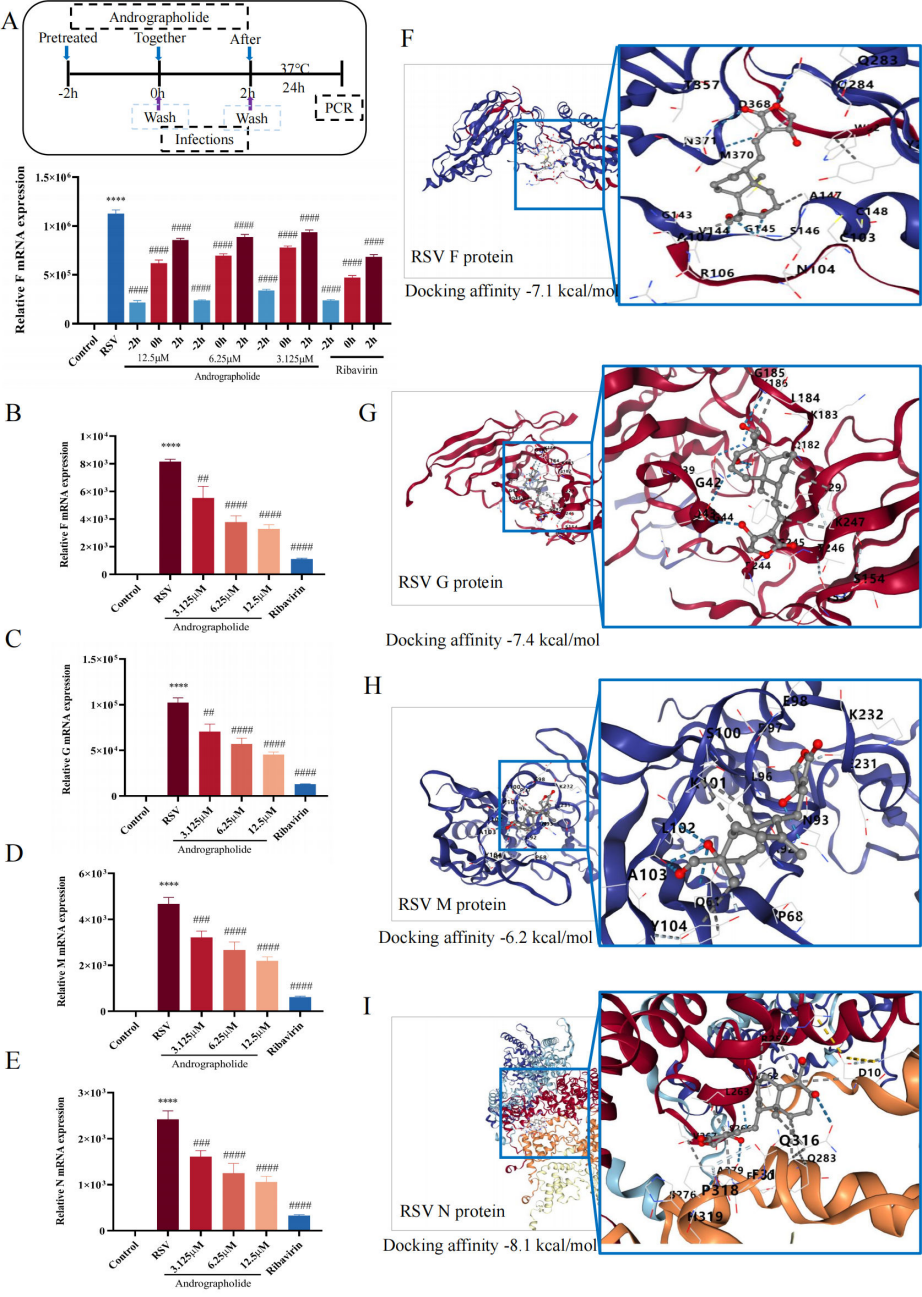
Time-of-addition assay and the influence of andrographolide on RSV proliferation cycle gene expression. (**A**) The scheme of time-of-addition assay and the inhibitory results of andrographolide on RSV F mRNA expression at different times (−2, 0, and 2 h) after RSV infection. (**B**) The effects of andrographolide on RSV F protein gene expression. (**C**) The effects of andrographolide on RSV G protein gene expression. (**D**) The effects of andrographolide on RSV M protein gene expression. (**E**) The effects of andrographolide on RSV N protein gene expression. (**F**) The molecular docking results of andrographolide with RSV F protein (PDBID: 7UJ3). (**G**) The molecular docking results of andrographolide with RSV G protein (PDBID: 5WN9). (**H**) The molecular docking results of andrographolide with RSV M protein (PDBID: 4D4T). (**I**) The molecular docking results of andrographolide with RSV N protein (PDBID: 8OP1). Student’s *t*-test was used for statistical analysis between the RSV group and the control group, while one-way ANOVA with Dunnett was used for statistical analysis of multiple groups. ****P < 0.0001 versus control group; ^####^*P* < 0.0001; ^###^*P* < 0.001; ^##^*P* < 0.01 versus RSV group.

As previously described (19), the RSV life cycle has some potential vulnerable points that can be targeted for the development of RSV therapeutics. Therefore, to determine the best administration time and potential antiviral mechanisms of andrographolide, the time-of-addition assay was carried out in this study. Figure 4A indicates that the administration of andrographolide (3.125, 6.25, and 12.5 μΜ) at −2, 0, or 2 h after RSV infections has significant inhibitory effects on the RSV F protein gene expression detected by real-time reverse transcription quantitative PCR (RT-qPCR). Ribavirin (6.25 µg/mL) also has the same inhibitory function. Importantly, before RSV infection, HEp-2 cells pretreated with andrographolide at −2 h showed the best inhibitory effects compared to ribavirin. Hence, these results suggest that andrographolide might affect the early stages of the virus lifecycle before viral entry.

To further ascertain the underlying antiviral mechanisms, the activities of andrographolide on the expression of RSV proliferation cycle-related protein genes, including RSV F, G, M, and N proteins, were assessed using RT-qPCR. The results demonstrated that andrographolide inhibited the gene expression of RSV F, G, M, and N proteins in a dose-dependent manner (Fig. 4B through D). Meanwhile, we further confirmed this result through molecular docking experiments. Figure 4F through I shows that the docking affinity of andrographolide with RSV F (PDBID: 7UJ3), G (PDBID: 5WN9), M (PDBID: 4D4T), and N (PDBID: 8OP1) was lower than −6.0 kcal/mol.

### Direct antiviral effects

**Fig 5:**
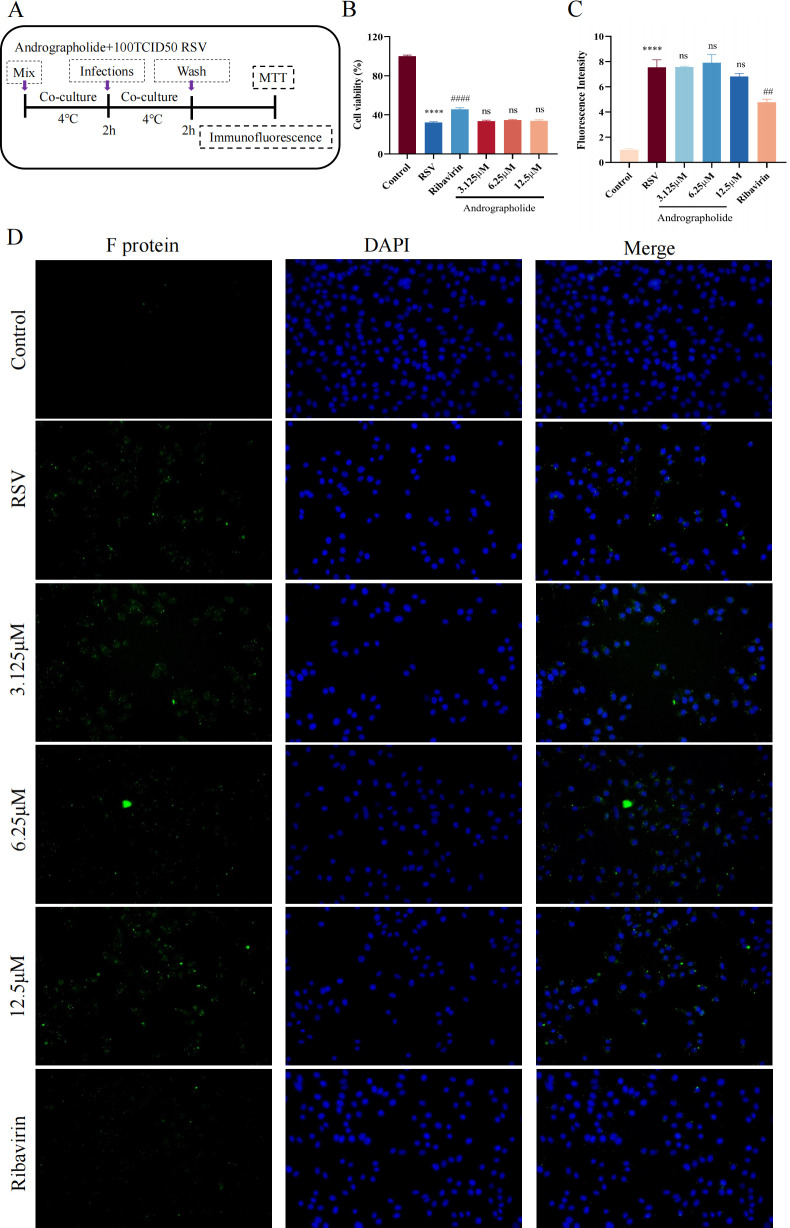
Cell viability and RSV F protein immunofluorescence detection on the direct antiviral effects of andrographolide on RSV. (**A**) Cell viability detection results of andrographolide on direct antiviral effects. (**B**) RSV F protein immunofluorescence results of andrographolide on direct antiviral effects. (**C**) Visualization of RSV F protein on the cell surface. (**D**) Merged photos show RSV F protein (green), DAPI (blue), and cells. Student’s *t*-test was used for statistical analysis between RSV group and control group, while one-way ANOVA with Dunnett was used for statistical analysis of multiple groups. ****P < 0.0001 versus control group; ^##^*P* < 0.01; ns, *P* > 0.05 versus RSV group.

Effective stage analysis was further performed to explore the mechanism of andrographolide on RSV and determine which stage of the RSV replication cycle andrographolide targets. We first investigated the viral inactivation effects of andrographolide by using cell viability and RSV F protein immunofluorescence detection. MTT assays showed that serial concentrations of andrographolide had no effect on the viability of cells infected by RSV (Fig. 5A). The immunofluorescence test results also indicated that andrographolide (3.125, 6.25, and 12.5 µM) has no significant effect on the expression of RSV F protein (Fig. 5B and C). On the contrary, ribavirin (6.25 µg/mL) has remarkable effects on cell viability and RSV F protein expression (Fig. 5). In short, these results indicate that andrographolide has no direct antiviral effects.

### Adsorption inhibition effect

**Fig 6:**
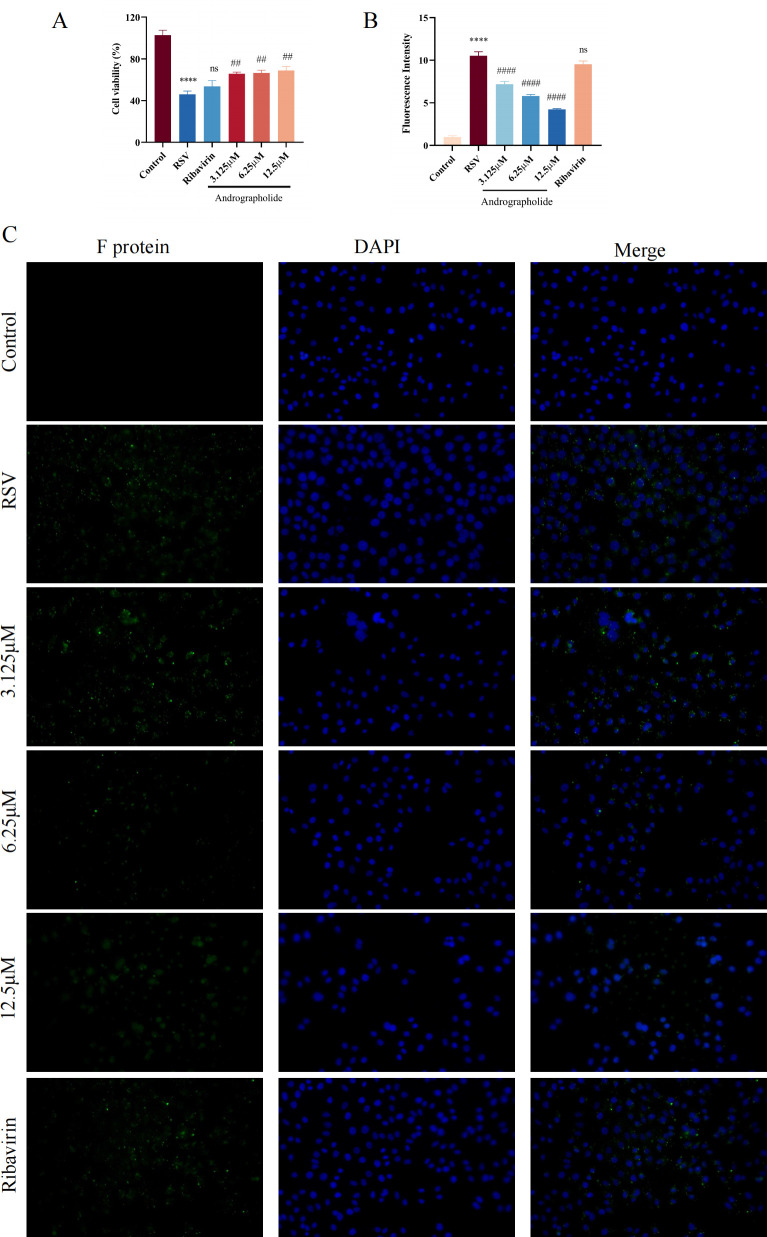
Cell viability and RSV F protein immunofluorescence detection on the adsorption inhibition effects of andrographolide on RSV. (**A**) Cell viability detection result of andrographolide on adsorption inhibition effects. (**B**) RSV F protein immunofluorescence results of andrographolide on adsorption inhibition effects. (**C**) Visualization of RSV F protein on the cell surface. Merged photos show RSV F protein (green), DAPI (blue), and cells. Student’s *t*-test was used for statistical analysis between the RSV group and the control group, while one-way ANOVA with Dunnett was used for statistical analysis of multiple groups. ^****^P < 0.0001 versus control group; ^####^*P* < 0.0001; ^##^*P* < 0.01; and ns, *P* > 0.05 versus RSV group.

The above results verified that andrographolide has no viral inactivation function. Then, the adsorption inhibition effect of andrographolide on RSV was further performed. Interestingly, we found that andrographolide (3.125, 6.25, and 12.5 µM) has significant inhibitory effects on the adsorption progress of RSV life cycling (Fig. 6A through C). Yet, ribavirin showed no significant adsorption inhibition effect (Fig. 6A through C). Based on this, it is speculated that the characteristic of andrographolide against RSV is to inhibit the process of RSV adsorption to the host, thereby exerting its anti-RSV infection effect.

### Replication and proliferation effect

**Fig 7:**
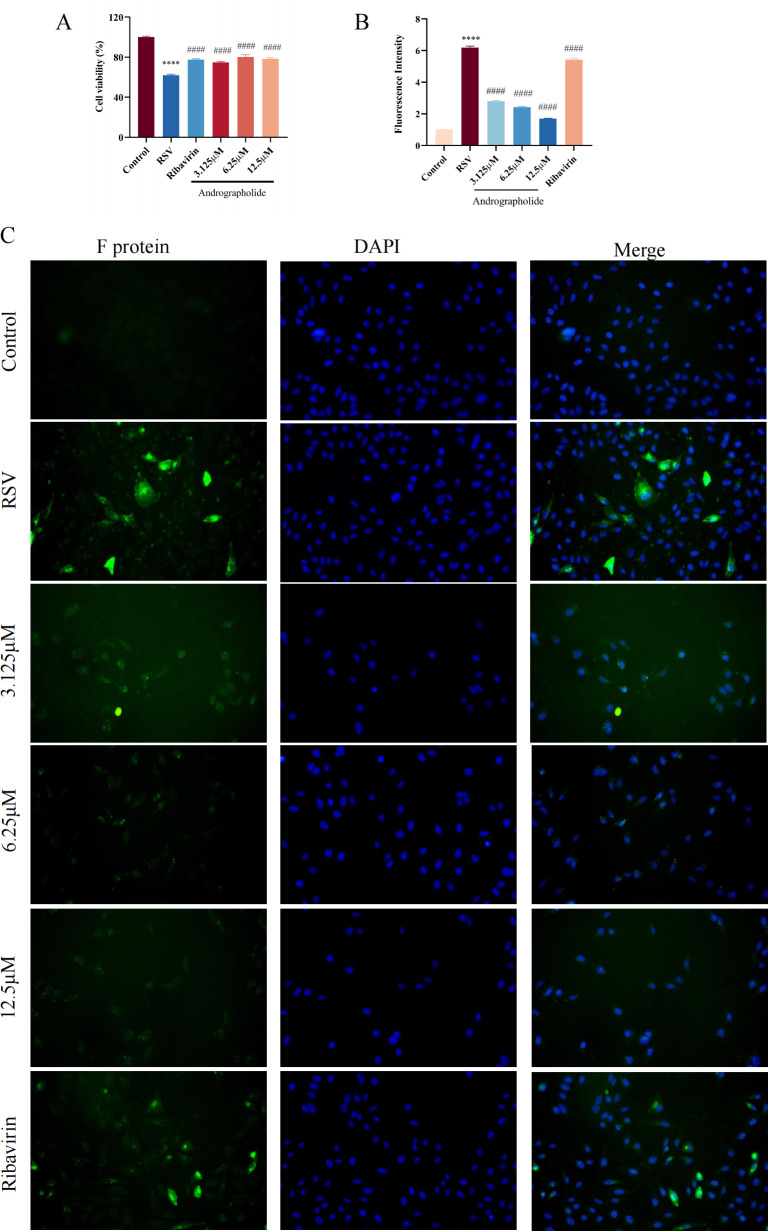
Cell viability and RSV F protein immunofluorescence detection on the replication and proliferation stage of andrographolide on RSV. (**A**) Cell viability detection result of andrographolide on replication and proliferation stage. (**B**) RSV F protein immunofluorescence results of andrographolide on replication and proliferation stage. (**C**) Visualization of RSV F protein on the cell surface. Merged photos show RSV F protein (green), DAPI (blue), and cells. Student’s *t*-test was used for statistical analysis between RSV group and control group, while one-way ANOVA with Dunnett was used for statistical analysis of multiple groups. ****P < 0.0001 versus control group; ^####^*P* < 0.0001 versus RSV group.

To further explore the effects of andrographolide on the replication and proliferation stage of RSV life cycle, we have also employed MTT assay and immunofluorescence test. The cell viability and immunofluorescence results indicate that both andrographolide (3.125, 6.25, and 12.5 µM) and ribavirin (6.25 µg/mL) have a dramatic inhibitory effect on RSV replication and proliferation stages (Fig. 7A through C). Taken together, the effects on the life cycle of RSV identified that andrographolide has a significant anti-RSV function.

Findings on the anti-RSV mechanisms demonstrate that andrographolide has significant and unique anti-RSV infection effects. Importantly, compared to ribavirin, the characteristics of andrographolide against RSV are possibly due to the inhibition of the adsorption process.

### Screening and verification of key adsorption receptors

The results revealed that the anti-RSV activities of andrographolide were characterized by inhibiting the adsorption stage of the RSV life cycle. Thus, the effects on known key adsorption receptors were screened via molecular docking and experiments and verified through CETSA and immunoblotting. The RSV-related adsorption receptors currently reported included CX3CR1 (20, 21), CD44 (22), and heparin-binding protein (HBP), as well as heparan sulfate proteoglycans (HSPGs) (23, 24). Then, we explored the interaction of andrographolide with these adsorption receptors mediating RSV infection.

**Fig 8:**
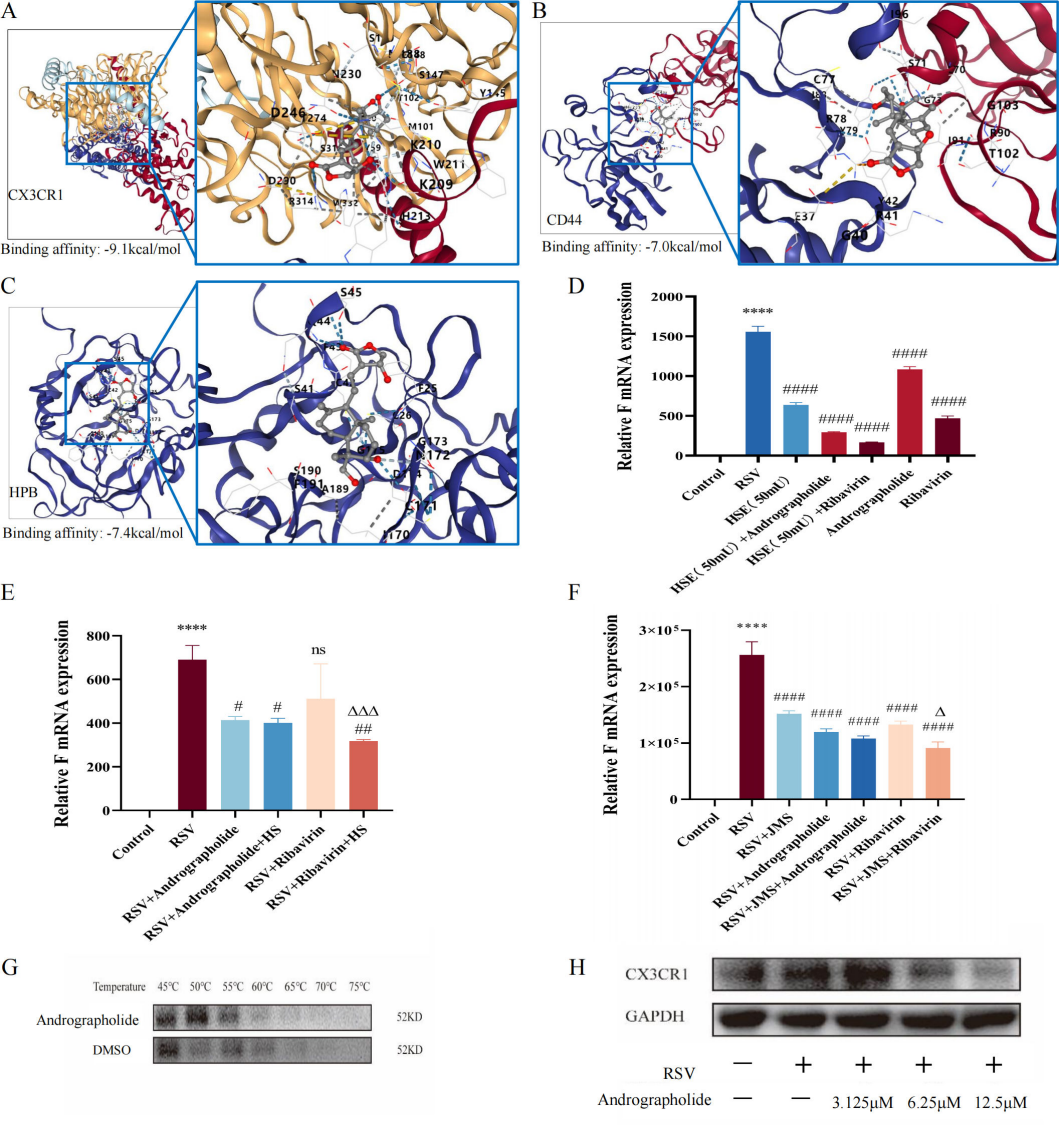
Screening and verification of key adsorption receptors of andrographolide during the adsorption process of the RSV life cycle. (**A**) Molecular docking prediction of andrographolide with CX3CR1 (PDB ID:7XBW, binding affinity: −9.1 kcal/mol). (**B**) Molecular docking prediction of andrographolide with CD44 (PDB ID:1UUH, binding affinity: −7.0 kcal/mol). (**C**) Molecular docking prediction of andrographolide with HBP (PDB ID:1AE5, binding affinity: −7.4 kcal/mol). (**D**) Virus load experiment on RSV F protein gene expression treated with andrographolide (12.5 µM), HSE, or their combination. (**E**) Virus load experiment on RSV F protein gene expression treated with andrographolide (12.5 µM), HS, or their combination. (**F**) Virus load experiment on RSV F protein gene expression treated with andrographolide (12.5 µM), JMS-17-2, or their combination. (**G**) Effects of andrographolide (12.5 µM) on the stability of CX3CR1 protein in cells with increasing temperature detected by CETSA. (**H**) Western blot verification on the effects of andrographolide (3.125, 6.25, and 12.5 µM) on CX3CR1. Student’s *t*-test was used for statistical analysis between RSV group and control group, RSV + ribavirin + HSE or HS or JMS group, and RSV + ribavirin group, while one-way ANOVA with Dunnett was used for statistical analysis of multiple groups. ****P < 0.0001 versus control group; ^####^*P* < 0.0001; ^##^*P* < 0.01; ^#^*P* < 0.05; and ns, *P* > 0.05 versus RSV group. ^△△△^*P* < 0.001 and ^△^*P* < 0.05 versus the ribavirin group.

Molecular docking results showed that andrographolide has high docking affinity with CX3CR1, CD44, and HBP. Most notably, the docking affinity with CX3CR1 was found to be the best (−9.1 kcal/mol) (Fig. 8A through C), indicating that the inhibitory effect of andrographolide on RSV adsorption may be mainly mediated by CX3CR1. Subsequently, we further tested the effects of andrographolide with heparan sulfate, heparinase, or JMS-17-2 (CX3CR1 inhibitor) on RSV F protein gene expression through viral load experiments, respectively. Findings in these results indicate that with or without the combination of heparan sulfate, heparinase, or JMS-17-2, andrographolide has significant inhibitory effects on the expression of RSV F protein gene (Fig. 8D through F).

However, the effects of andrographolide on CX3CR1 expression still need to be investigated. Subsequently, CETSA was performed, and we discovered that, compared with the DMSO treatment group, the intervention of andrographolide could significantly increase the stability of CX3CR1 protein in cells with increasing temperature (Fig. 8G). Finally, Western blot analysis verified that andrographolide can significantly inhibit the expression of CX3CR1 protein (Fig. 8H).

### Network pharmacological analysis results

Our research findings indicate that the early anti-RSV effect of andrographolide is achieved through competitive inhibition of RSV binding to CX3CR1. However, it is necessary to further elucidate how andrographolide affects the mechanism by which hosts exert their resistance against RSV infection. Hence, network pharmacology-based analysis was performed to unveil the underlying anti-RSV mechanisms of andrographolide.

**Fig 9:**
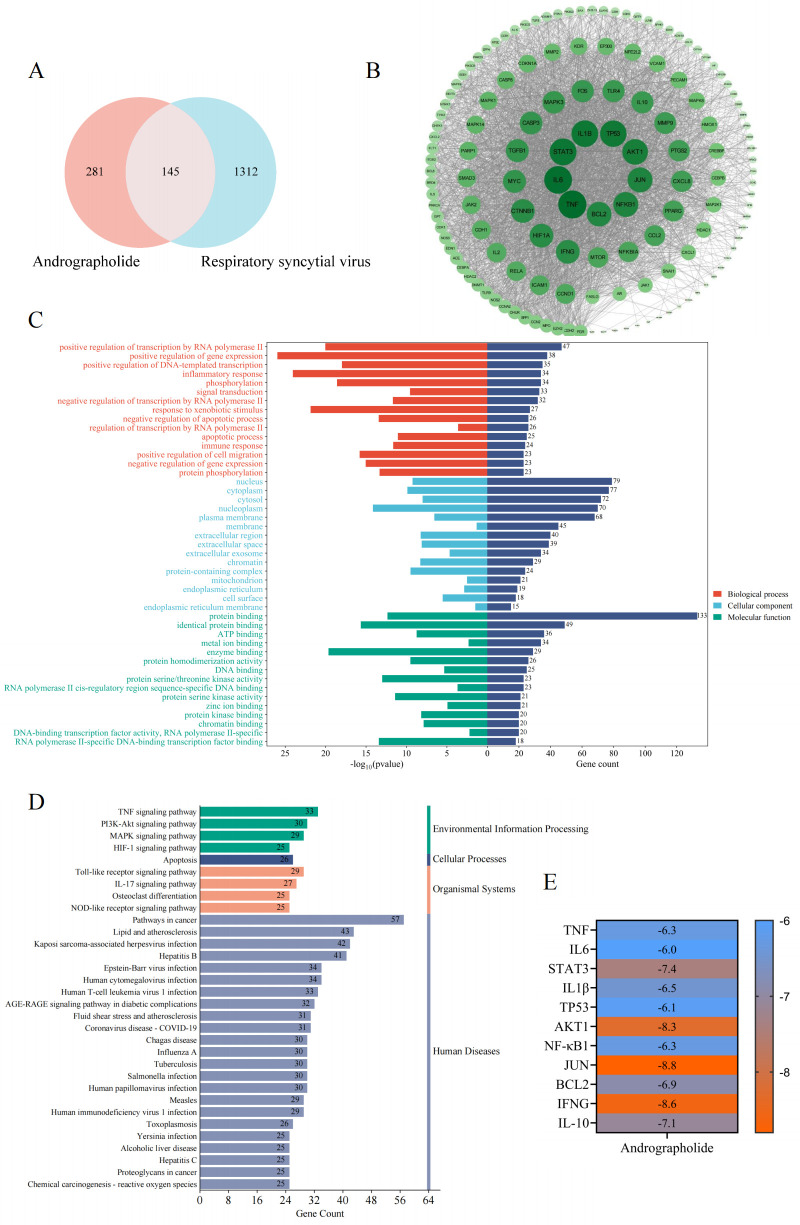
Network pharmacology-based analysis reveals the anti-RSV mechanisms of andrographolide. (**A**) Venn diagram of the potential therapeutic targets of andrographolide in treating RSV. (**B**) PPI network and the core target identification. (**C**) Visualization of the GO function enrichment, including biological process, cellular component, and molecular function. (**D**) Visualization of the Kyoto Encyclopedia of Genes and Genomes pathway enrichment. (**E**) Molecular docking affinity results of andrographolide with the core therapeutic targets.

Through analysis, a total of 426 targets were predicted as the potential targets of andrographolide, and 1,457 targets were considered as the therapeutic targets of RSV, after standardizing and removing the duplicate values. Further analysis showed that a total of 145 targets, including TNF, NF-κB, STAT3, IL6, IL1B, AKT1, JUN, and BCL2, were identified as the latent anti-RSV therapeutic targets of andrographolide (Fig. 9A). To further explore the core targets of andrographolide against RSV, we constructed a protein-protein interaction (PPI) network and found that the top 10 targets were mainly TNF, NF-κB, STAT3, IL6, IL1B, AKT1, JUN, TP53, and BCL2, as well as IFNG, based on degree value analysis (Fig. 9B). Gene Ontology (GO) analysis showed that treatment of RSV with andrographolide may affect the positive regulation of gene expression, inflammatory response, phosphorylation, signal transduction, response to exogenous stimuli, and negative regulation of apoptosis of biological processes; affect the nucleus, cytoplasm, and extracellular secretion of cellular components; and affect the protein binding, ATP binding, and enzyme binding of molecular functions (Fig. 9C). The antivirus mechanism of andrographolide may involve modulating the TNF, PI3K-Akt, MAPK, HIF1A, apoptosis, and toll-like receptor signaling pathways (Fig. 9D). Molecular docking indicated that andrographolide has a higher binding affinity with the key targets, with binding affinities all lower than −5.0 kcal/mol (Fig. 9E).

### Validation of the ROS/TXNIP/NF-κB pathway based on current key findings

**Fig 10:**
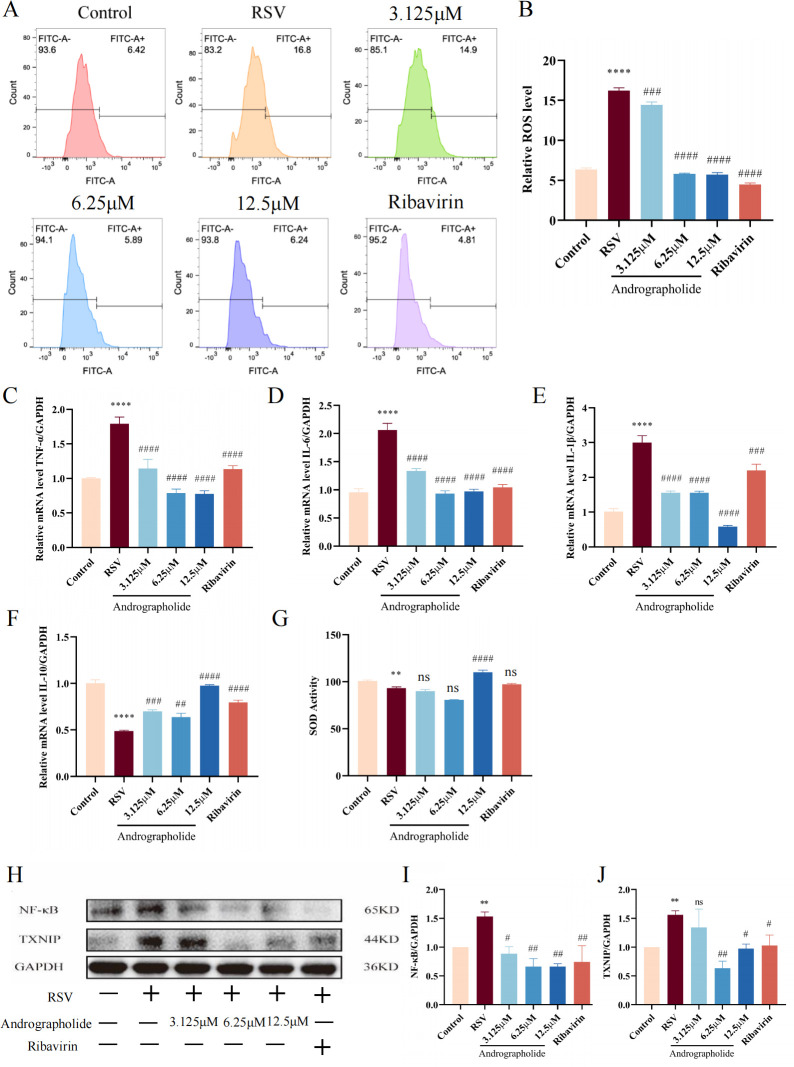
Validation of the modulating effects of andrographolide on ROS, inflammatory mediators, and the ROS/TXNIP/NF-κB pathway based on current key findings. (**A**) ROS detection based on flow cytometry. (**B**) Visualization of ROS quantitative analysis results based on flow cytometry. (**C**) PCR detection results of andrographolide on TNF-α expression. (**D**) PCR detection results of andrographolide on IL-6 expression. (**E**) PCR detection results of andrographolide on IL-1β expression. (**F**) PCR detection results of andrographolide on IL-10 expression. (**G**) ELISA detection results of andrographolide on SOD expression. (**H**) Western blot analysis of andrographolide on TXNIP and NF-κB expression. (**I**) Visualization of Western blot quantitative analysis results of NF-κB. (**J**) Visualization of Western blot quantitative analysis results of TXNIP. Student’s *t*-test was used for statistical analysis between RSV group and control group, while one-way ANOVA with Dunnett was used for statistical analysis of multiple groups. ****P < 0.0001; **P < 0.01 versus control group; ^####^*P* < 0.0001; ^###^*P* < 0.001; ^##^*P* < 0.01; ^#^*P* < 0.05; ns, *P* > 0.05 versus RSV group.

Based on the current findings, we hypothesized that andrographolide mainly binds to the RSV adsorption receptor CX3CR1, thus competitively inhibiting the RSV adsorption process, and CX3CR1 mediates oxidative stress and inflammatory response. We utilized flow cytometry to test the expression of reactive oxygen species (ROS) and discovered that andrographolide (3.125, 6.25, and 12.5 µM) has significant inhibitory effect on ROS after infected by RSV (Fig. 10A and B), suggesting that andrographolide has significant antioxidant stress effects. PCR detection results further show that andrographolide dramatically reduced the inflammatory factors of TNF-α, IL-6, and IL-1β (Fig. 10C through E) and significantly increased the anti-inflammatory mediator of IL-10 (Fig. 10F) after the infection of RSV. Meanwhile, we also used enzyme-linked immunosorbent assay (ELISA) kits to investigate the effects of andrographolide on superoxide dismutase (SOD). To an extent, andrographolide can increase the expression of SOD (Fig. 10G). Finally, the upstream targets regulating ROS and inflammatory response, including TXNIP and NF-κB, were detected by immunoblotting analysis. The results suggested that andrographolide can decrease the protein expression of TXNIP and NF-κB induced by RSV infection (Fig. 10H through J).

## DISCUSSION

RSV-induced ALRTs, a global public health challenge, severely affect infants and children under 5 years old, the elderly, immunodeficient patients, causing a substantial burden on patients’ family and healthcare system (25). Despite considerable research efforts (9, 26), patients’ awareness of the burden and perniciousness of RSV infection and effective drug therapies for RSV-induced ALRTs remain limited. In the current study, we first analyzed the global epidemiology and burdens caused by RSV infections using data from the GBD database. It is found that the overall RSV mortality and DALY rates have decreased globally from 1990 to 2021, attributing to the development of the economy and society, as well as the implementation of disease prevention and control measures. However, children and the elderly remain high-risk populations (5, 27), and in low SDI regions with relatively backward economic and cultural levels such as South Africa (28), Sub-Saharan Africa (29), and Latin America (30), or other resource-constrained settings (31), the burden of RSV disease is still relatively severe. More importantly, our findings indicate that the economic burdens and harms caused by RSV infection cannot be ignored, and intervention measures still need to be taken.

Meanwhile, due to the limited acquired and appropriate agents, the treatment of the infants, children (aged <5), and the elderly with RSV infection are insufficient. Hence, we propose the development of andrographolide as a promising anti-RSV drug from the perspective of “drug repositioning.” Andrographolide, a marketed drug, has been applied in clinics for many years. Previous studies highlighted andrographolide’s anti-inflammatory (32), antineoplastic (33), and antiviral properties (34), with reports indicating its anti-RSV ability *in vitro* and *in vivo* (35, 36). However, the anti-RSV characteristics of andrographolide have not been revealed, hindering its further development and use in the treatment of RSV infection. To unveil the anti-RSV characteristics and underlying mechanism of andrographolide, we first investigated its effects on the expression of RSV F, G, M, and N protein genes during the RSV life cycle. It is generally agreed that RSV, an RNA virus with various shapes such as spherical and filamentous, contains a genome of 15,222 nucleotides of non-segmented negative-stranded RNA (37), encoding NS1, NS2, N, P, M, SH, G, F, M2-1, M2-2, and L proteins (38). Among them, M and M2 are envelope proteins, RSV F and RSV G proteins are glycoproteins, and SH is a type of small hydrophobic protein (39). Research has confirmed that RSV G protein, a highly glycosylated protein, is responsible for virus adsorption to the host, while RSV F protein mediates the fusion process between the virus and the host. RSV N protein is a protein that wraps around and protects the viral genome, which is essential for viral transcription and replication. On the contrary, RSV M protein plays a key role in viral assembly and post-infection budding (37, 40). Our results showed that andrographolide possesses good binding affinities and inhibitory effects on the RSV F, G, M, and N protein gene expression (Fig. 4), indicating that andrographolide could be considered a promising anti-RSV drug.

In the following antiviral mechanism experiments, we innovatively discovered that the inhibitory effects of andrographolide on virus adsorption process during RSV life cycle are better than ribavirin in RSV-infected HEp-2 cell models (Fig. 6). Subsequently, we further investigated the effect of andrographolide on known RSV adsorption-related receptors. RSV G protein consists of a cytoplasmic domain at the N-terminus, a transmembrane domain, and an extracellular domain at the carboxy terminus. The extracellular domain includes two highly glycosylated variable mucin-like domains at both ends and a central conserved domain (CCD) with four cysteine residues in the central sequence (41, 42). While the CCD is highly conserved, containing a conserved region consisting of 13 amino acids, a CX3C chemokine motif, and a heparin binding domain (HBD) (24). In addition, the positively charged HBD in RSV G protein also facilitates RS virus attachment to the host through cell surface glycosaminoglycans (GAGs) (24, 43). Currently, GAGs mainly include hyaluronic acid (HA), heparin (HP)/heparan sulfate (HS), chondroitin sulfate (CS)/dermatan sulfate (DS), and keratin sulfate (KS). Collectively, G protein mainly acts on chemokine receptor 1 (CX3CR1) in host receptors and cell surface GAGs, thereby mediating the occurrence of RSV infection. Therefore, the RSV G protein or CX3CR1 can be targeted for new drug developments. We found that andrographolide has certain inhibitory function on HSE, HS, and CX3CR1 (Fig. 8). However, there is still some controversy regarding the application of HSPG analogs or inhibitors as anti-RSV agents. Hence, we speculated that andrographolide affects RSV adsorption to the host by competitively binding to the CX3CR1 receptor with RSV G protein. Further CSETA tests proved that andrographolide could significantly increase the stability of CX3CR1 protein (Fig. 8). These findings proclaimed that the anti-RSV characteristics of andrographolide lay in inhibiting the RS virus adsorption to the host in the early stage of RSV life cycling.

Subsequently, we further investigated the antiviral mechanism of andrographolide against CX3CR1-mediated RSV infection in hosts by integrating network pharmacological analysis and molecular biology experiments. CX3CR1 plays an important role in the adhesion and migration of white blood cells. The upregulation of CX3CR1 expression in inflammatory cells enhances the aggregation of inflammatory cells and participates in the pathological development of inflammatory diseases (44). Gan et al. (45) found that activation of the NF-κB/AP-1/STAT1/STAT3 pathway can upregulate the expression of CX3CL1/CX3CR1, thereby promoting inflammation in smooth muscle cells. In addition, the binding of IL-1β to IL-1R1 can promote the expression of CX3CR1 in chronic inflammatory animal models (46). Therefore, activation of inflammatory signaling pathways (such as TLR4 and NF-κB) promotes high expression of CX3CR1. Moreover, studies have confirmed a positive correlation between oxidative stress levels and inflammatory responses, and CX3CR1 exacerbates oxidative stress damage in NCTC1469 cells by inhibiting the JAK2/STAT3 signaling pathway (47). These suggest that the downstream pathway mediated by CX3CR1 is closely related to oxidative stress and cellular inflammatory response.

In PPI analysis, we screened TNF, IL-6, STAT3, IL-1β, AKT1, NF-κB1, and IL-10 as the core targets of andrographolide against RSV infection (Fig. 9B). Among them, IL-6, IL-1β, NF-κB, and IL-10 are closely related to inflammation and anti-inflammatory response, while SOD1 and catalase (CAT) are closely related to antioxidant stress response. GO enrichment analysis results indicated that the main biological processes involved are inflammatory reactions (Fig. 9C). The major anti-RSV pathways of andrographolide include TNF, TLR, IL-17, NOD-like receptor (NLR), and NF-κB signaling pathway (Fig. 9D). These results suggest that andrographolide can inhibit inflammatory response and oxidative stress caused by RS virus infection. Activation of the NF-κB pathway (48) induces cellular responses such as cell survival, differentiation, cell proliferation, immune response, and inflammation (49). Andrographolide closely docks with TNF, IL-6, STAT3, IL-1β, AKT1, NF-κB1, and IL-10 (Fig. 9E). RT-qPCR analysis identified that andrographolide significantly inhibited the gene expression of inflammatory factors (TNF-α, IL-6, and IL-1β) after RSV infection (Fig. 10C through E), while simultaneously increasing the expression of the anti-inflammatory cytokine IL-10 (Fig. 10F). Research has confirmed that during RSV infection, RSV F protein binds to the host TLR4 receptor, promoting virus invasion and activating downstream NF-κB (50), suggesting that NF-κB may be the main activating factor of the innate immune response in RSV infection (51). Immunoblotting analysis showed that andrographolide markedly decreased the NF-κB protein expression (Fig. 10H and I). Zhou et al. (35) also demonstrated that intranasal andrographolide sulfonate significantly downregulated RSV N and RSV F proteins, TLR3, and TRIF protein expression in the lung to ameliorate lung inflammation in RSV-infected animals. In addition, andrographolide has been proven to inhibit the activation of the NF-κB pathway induced by lipopolysaccharide (LPS) (52) and exert potent anti-inflammatory and anti-ferroptosis effects in LPS-induced RAW 264.7 cells and acute lung injury by targeting TLR4 and modulating the Keap1/Nrf2 pathway (53). Our study findings and the reported evidence validate that andrographolide has a potent anti-inflammatory response to RSV infection.

**Fig 11:**
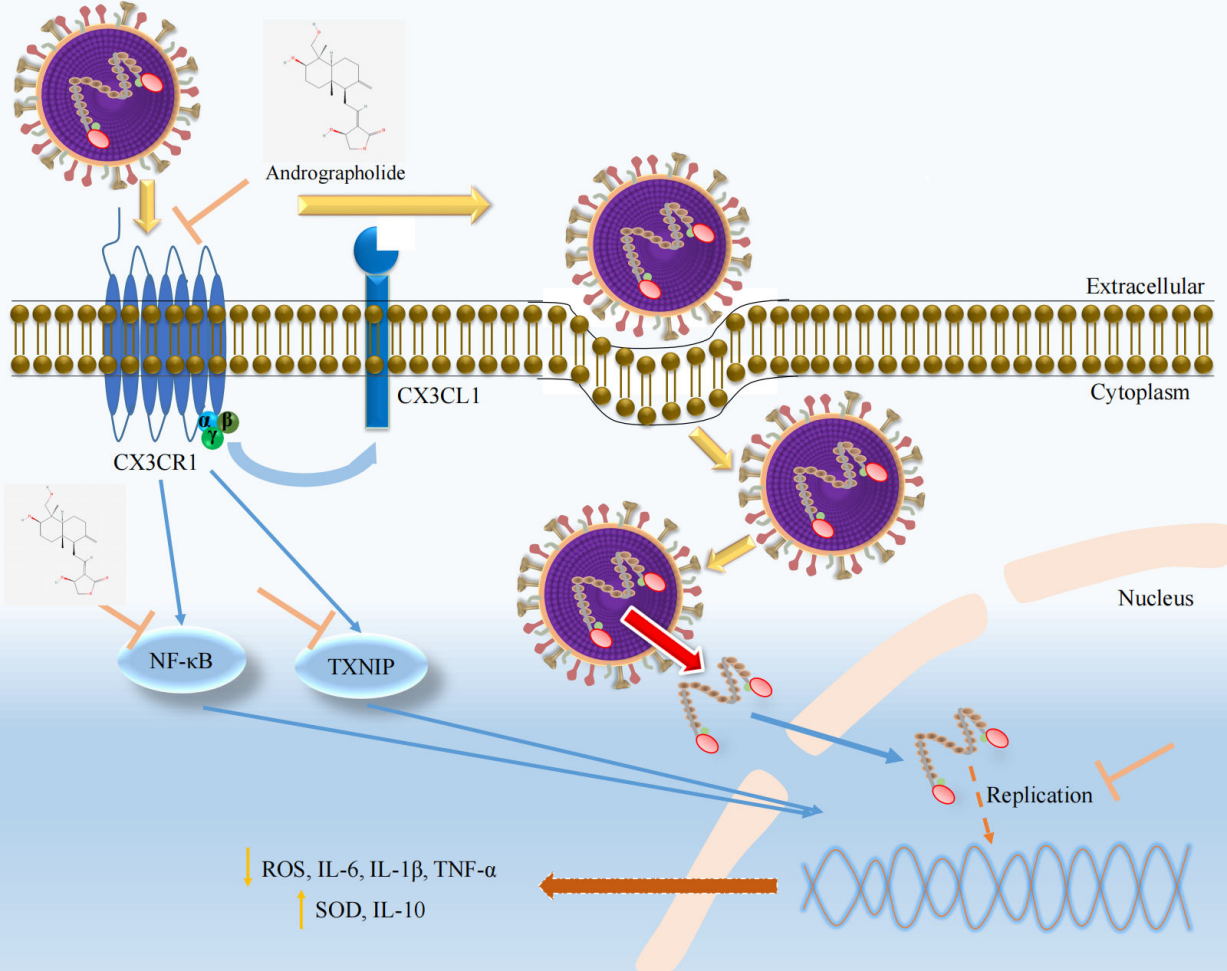
Mechanism of andrographolide against respiratory syncytial virus infection. Andrographolide competitively inhibits viral binding to host cell receptors, mainly the CX3CR1 receptor, thereby blocking its interaction with the host cell and preventing infection. It also inhibits activation of the ROS/NF-κB/TXNIP pathway mediated by CX3CR1 induced by respiratory syncytial virus infection, thereby inhibiting oxidative stress and inflammatory responses.

During PPI analysis, we observed that SOD1 and CAT are related to oxidative stress and have a close relationship between oxidative stress and inflammatory response (54). After RSV infection, it leads to the production of oxygen free radicals, while clearing ROS or inhibiting intracellular ROS levels can significantly reduce the production of inflammatory mediators (55). Accumulating evidence suggests that the expression levels of ROS and SOD in the body have undergone significant changes after RSV infection (56, 57). In addition, the severity of acute bronchiolitis caused by RSV infection is related to oxidative stress (58). Therefore, we concurrently investigate the effect of andrographolide on the expression of ROS and SOD. Flow cytometry analysis revealed that andrographolide remarkably decreased the ROS levels induced by RSV infection (Fig. 10A and B). The ELISA results also indicate that andrographolide increased the level of SOD to a certain extent (Fig. 10G). These key findings suggest that andrographolide can inhibit the oxidative stress response caused by RSV infection. We noticed that thioredoxin interacting protein (TXNIP), an oxidative stress regulator involved in cell proliferation, differentiation, and apoptosis, is a protein that connects oxidative stress and inflammasome activation (59, 60). It can interact with thioredoxin (TRX) in the cytoplasm and mitochondria, ultimately activating NLRP3 inflammasome (61). Therefore, TXNIP is regarded as an endogenous negative regulator of TRX1 and a key pathological regulator of various diseases, making it a potential therapeutic target for new drug development (60). In our study, we detected the efficacy of andrographolide on TXNIP and clarified that andrographolide significantly suppresses the expression of TXNIP protein (Fig. 10H and J). Collectively, our results preliminarily confirm that andrographolide regulates the ROS/TXNIP/NF-κB signaling pathway through CX3CR1, thereby inhibiting oxidative stress and inflammatory processes and exerting a comprehensive therapeutic effect against RSV infection (Fig. 11).

Taken together, the findings of this work suggest that people should pay attention to the severe burdens and serious harms that RSV infection brings to families and society worldwide. It also provides new insights into the clinical application of andrographolide, a promising drug, for the treatment of RSV infection. However, this study also has shortcomings. For instance, further experiments are needed to supplement and validate the screening results of the key adsorption receptors of andrographolide against RSV. Second, our study did not conduct *in vivo* animal experiments simultaneously to further reveal the regulatory mechanism of andrographolide on the ROS/TXNIP/NF-κB signaling pathway mediated by CX3CR1. Multiple experimental studies are still needed in the future to clarify the current key findings highlighted.

### Conclusions

Collectively, in this study, we highlighted that the worldwide disease burdens induced by RSV infection are still severe, especially in economically underdeveloped countries or regions. Infants, young children, and the elderly remain the high-risk population. Meanwhile, a new therapeutic strategy for the treatment of RSV infection was proposed, and andrographolide was identified for the first time as a competitive inhibitor of RSV adsorption to host cells via the CX3CR1 receptor. Further antiviral mechanism experiments proved that andrographolide suppresses oxidative stress and inflammatory responses mediated by the inactivation of the ROS/TXNIP/NF-κB signaling pathway, thereby exerting its anti-RSV efficacy. Overall, key findings in our study provide new insight into the prevention and treatment of RSV infection and scientific basis for developing andrographolide as a new drug for treating RSV infection.

## MATERIALS AND METHODS

### GBD database-based epidemiological trends of RSV infection analysis

The data are sourced from the GBD 2021 database, an open access database that provides the latest comprehensive standardized estimates of epidemiological data for 371 diseases and injuries, 288 causes of death, and 88 risk factors in different genders and age groups across 21 geographic regions, 204 countries, and 811 subnational regions from 1990 to 2021 (62). In this study, classification was conducted based on the GBD 2021 database by year (1990–2021), gender (male/female), age (20 age groups), geographic location, and socio-demographic index (SDI) (63). The number of deaths, disability-adjusted life years, and age-standardized rates of ALRTI caused by RSV from 1990 to 2021 were retrieved. According to SDI values, 204 countries or regions are divided into five SDI groups: low, medium-low, medium, medium-high, and high SDI regions. Based on epidemiological similarity and geographical proximity, we further divide 204 countries (regions) into 21 GBD regions.

A total of 19 age groups were divided by 5 years per group, including “under 5 years old,” “5–9 years old,” “10–14 years old,” “15–19 years old,” “20–24 years old,” “25–29 years old,” “30–34 years old,” “35–39 years old,” “40–44 years old,” “45–49 years old,” “50–54 years old,” “55–59 years old,” “60–64 years old,” “65–69 years old,” “70–74 years old,” “75–79 years old,” “80–84 years old,” “85–89 years old,” “90–94 years old,” and “95 years old.”

To evaluate the influences and epidemiological burdens caused by RSV infection, both mortality and disability-adjusted life years were used as evaluation indicators. Meanwhile, the Joinpoint regression model (JRM) was used to analyze the time trends of age-standardized mortality rate and age-standardized rate DALYs caused by RSV infection from 1992 to 2021. The average annual percent change, annual percent change (APC), and their 95% confidence intervals were calculated. The fitting of JRM and the calculation of AAPC and APC were completed by R software (version 4.4.1) and visualized using Joinpoint software, with a test level set at *α* = 0.05. In addition, the age period cohort model is used to analyze the effects of age, period, and birth cohort on RSV mortality and DALYs. The queue effect reveals the influence of different socio-economic, environmental risks, dietary habits, and other factors on populations born at different stages. The calculation was completed using R software (version 4.4.1) and the Age Period Cohort Analysis Tool was used (https://analysistools.cancer.gov/apc/) for visualization. Finally, the ARIMA model was employed to predict the RSV disease burden until 2034, which was calculated and visualized by R software (version 4.4.1).

### Drug and reagents

Andrographolide (lot: 41221103) was provided by Lizhu Group Limin Pharmaceutical Factory (Zhuhai, Guangdong province, China). Ribavirin capsules (lot: 221201) were purchased from Guangdong South China Pharmaceutical Group Co., Ltd. (Dongguan, Guangdong province, China). Dulbecco’s modified Eagle’s medium (DMEM, containing dual antibodies, lot: 20231213), trypsin EDTA digestion solution (lot: 20231227), 10× phosphate-buffered saline (PBS, lot: 20231231), BSA (Standard Grade, lot: 20240507), dimethyl sulfoxide (cell level, DMSO, lot: 20240108), RIPA cracking solution (lot: 20240905), PMSF (lot: 20230306), and phosphatase inhibitor mixture (Cocktail, lot: 20230505) were obtained from KeyGEN BioTECH (Nanjing, Jiangsu Province, China). Fetal bovine serum (FBS, cat: 12B326) was purchased from ExCell Bio. (Suzhou, Jiangsu Province, China). Steady Pure RNA Extraction Kit (without lysis buffer, lot: A5A3860) and AG RNAex Pro reagent (lot: A5A4070) were purchased from AG Accurate Biology (Changsha, Hunan province, China).

HiScript III All-in-one RT SuperMix Perfect for qPCR (lot: 7E731J3), ChamQ Blue Universal SYBR qPCR Master Mix (lot: 7E781G3), and 180 kDa Prestained Protein Marker (lot: 7E652G2) were obtained from Vazyme Biotech (Nanjing, Jiangsu province, China). MTT powder (3-(4,5-Dimethylthiazol-2-yl)−2,5-diphenyltetrazolium bromide) (lot: EZ7890D473) and nonfat dried milk (lot: EZ6789D104) were purchased from BioFroxx (Germany). RSV F protein antibody (lot: 23666) was purchased from AntibodySystem (France). Ready to use normal goat serum (lot: 20240701) and total superoxide dismutase assay kit (WST-1 Method) were obtained from Nanjing Jiancheng Bioengineering Research Institute (Nanjing, Jiangsu province, China). FITC-labeled goat anti-mouse IgG (H + L) (lot: 062723240521), DAPI (lot: 041923230928), and primary antibody diluent (lot: A046241024) were purchased from Beyotime Biotechnology (Shanghai, China). Triton X-100 (lot: T109027) was purchased from Aladdin (Shanghai, China). CX3CR1 antibody (lot: 5500005732) was purchased from ABclonal Biotechnology Co., Ltd. (Wuhan, Hubei province, China). JMS-17-2 (lot: 50424) and heparan sulfate (lot: 26834) were provided by MedChemExpress. Heparinase III derived from Heparin Yellow Bacteria (lot: 0000369562) was provided by Sigma-Aldrich (Germany).

### Cell culture and viral pathogenicity assay

Cells culture and RSV amplification culture were performed as previously described (64, 65). HEp-2 cell lines were purchased from FUBIO (Future Biotechnology Co., Ltd., Suzhou, Jiangsu province, China) and were incubated in DMEM supplemented with 10% FBS at 37°C in an atmosphere containing 5% CO_2_. The human respiratory syncytial virus (strain A2 Long, from China Center for Type Culture Collection, Wuhan, China) was generously gifted by Prof. Bin Yuan of Jiangsu Key Laboratory of Pediatric Respiratory Disease, Jiangsu Province Hospital of Chinese Medicine, Nanjing University of Chinese Medicine (66, 67). As described previously (68, 69), the RSV amplification was primarily carried out using HEp-2 cells, and the TCID50 (Median Tissue Culture Infectious Dose) assay was employed to detect the viral titers. HEp-2 cells, housed in a 96-well plate, were exposed to serial dilutions of the RSV. After an incubation period of 72 h, cellular lesions within each well were assessed under the microscope. Wells were denoted as RSV infected when over 50% of its cells exhibited pathogenic signs. Viral titers, defined as TCID50, were ascertained via the Reed–Muench method. During viral infections, DMEM containing 2% FBS was utilized, and any unattached viruses were meticulously washed off twice using PBS.

### Cytotoxicity assay

HEp-2 cells were transferred to 96-well plates at a rate of 1 × 10^5^ cells per well. After the cells were attached to the wall, different concentrations of andrographolide or ribavirin were added to the cells and continually incubated for 24 h. The viability of the cells was detected via the MTT method (5 mg/mL, 0.25 g MTT in 50 mL of 1× PBS). The cell survival rate was determined based on the absorbance of the cells in each well determined by Multiskan Spectrum microplate reader (Multiskan GO, Thermo Fisher Scientific [Shanghai] Instrument Co., Ltd).

### Time-of-addition assay

To determine the optimal administration time of drugs, a time-of-addition assay was performed modified according to the previous study (19). Briefly, HEp-2 cells were seeded into a 96-well plate and infected with 100 TCID_50_ RSV per well. These cells were treated with andrographolide or ribavirin solution at −2, 0, and 2 h post-RSV infection. When the cytopathic effect of the RSV control group reached 90%, the incubation was stopped, and total RNA was collected from the cell samples. Then, the RT-PCR method was utilized to test the RSV F protein gene expression level.

### Effects on RSV lifecycle-related proteins

Before conducting research on antiviral mechanisms, the effects of andrographolide on RSV lifecycle-related proteins were investigated. HEp-2 cells cultured in six plates were pretreated with different concentrations of andrographolide (3.125, 6.25, and 12.5 µM) or ribavirin (6.25 µg/mL) for 2 h at 37°C, then 100 TCID_50_ RSV was added and co-incubated for 24 h. After 24 h, the total RNA was extracted, and the expression levels of RSV F, G, M, and N proteins were detected by RT-qPCR.

### Antiviral mechanisms

#### Direct virucidal action

Both MTT and immunofluorescence methods were utilized to evaluate the direct virucidal action of andrographolide. The 96-well plate inoculated with HEp-2 cells was pre-cooled at 4°C for 1 h. First, different concentrations of andrographolide (3.125, 6.25, and 12.5 µM) and ribavirin (6.25 µg/mL) solutions were mixed with equal volumes of RSV solution (100 TCID_50_), and the drug-RSV mixture was co-cultured at 4°C for 2 h. Then, the drug-virus mixture was added to the pre-cooled 96-well plate and incubated at 4°C for 2 h. The supernatant was removed and washed three times with 1× PBS. Finally, 100 µL of DMEM was added, with six wells set up for each concentration, along with a control group and a virus group. The MTT assay was performed until the RSV group cells had an infection rate of 75%. The immunofluorescence test was carried out after finishing the co-incubation at 4°C for 2 h.

#### Inhibition of viral adsorption

Serial concentration of andrographolide (3.125, 6.25, and 12.5 µM) and ribavirin (6.25 µg/mL) solutions were mixed with 100 TCID_50_ RSV and co-incubated for 2 h at 4°C. Subsequently, the drug-virus mixture was inoculated into HEp-2 cells pre-cooled for 1 h in advance and cultured at 4°C for 2 h. The supernatant was then discarded and washed three times with 1× PBS, followed by the addition of 100 µL DMEM medium. Each group had six replicates, and both the control group and the virus group were established. The MTT test was employed until the RSV group had an infection rate of 75%. In parallel, after the virus had fully adsorbed to the host cell at 4°C for 2 h, the immunofluorescence experiment was performed.

#### Inhibition of viral replication and proliferation

After pre-cooling the HEp-2 cells inoculated in a 96-well plate for 1 h, the supernatant was discarded, and 100 TCID50 RSV solution was added. Then, the cells were incubated at 4°C to allow RSV to adsorb onto the cell membrane surface without internalization. After 2 h, the virus solution was discarded, and andrographolide (3.125, 6.25, and 12.5 µM) and ribavirin (6.25 µg/mL) were added and incubated in a 37°C, 5% CO_2_ constant temperature incubator until the RSV group cell infection rate reached 75%. The MTT assay was used to detect cell survival rate. An immunofluorescence experiment was utilized to investigate the inhibition effects on RSV replication and proliferation.

### Screening and identification of the effect of andrographolide on key RSV adsorption receptors

The aforementioned antiviral mechanism results found that the characteristic of andrographolide in resisting RSV is to inhibit RSV adsorption to the host. RSV G protein, an adsorption receptor protein, has been shown to bind to host cell surface receptor CX3CR1 (21) or heparan sulfate proteoglycans in glycosaminoglycan, promoting RSV adsorption and leading to host cell infection (38, 70).

To screen and identify the effect of andrographolide on key RSV adsorption receptors mentioned above, we first utilized molecular docking to explore the potential binding affinity of andrographolide with known RSV adsorption receptors. Then, a viral load test was performed further to identify the combination of andrographolide with heparan sulfate, heparinase, or JMS-17-2 (CX3CR1 inhibitor) on RSV F protein mRNA expression. In brief, 1 µL of 5 mg/mL heparan sulfate was dissolved in 1 mL of pre-prepared andrographolide (12.5 µM) or ribavirin (6.25 µg/mL) solution, mixed well, and incubated at 4°C for 30 min. Then, the mixture was added to a 6-well plate of HEp-2 cells and incubated at 37°C for 2 h. Subsequently, RSV (MOI = 1) was added and incubated in a 37°C, 5% CO_2_ incubator for 24 h. Finally, the total RNA of the cells was extracted, and the viral load (RSV F protein gene expression) was detected by RT-qPCR. Before adding andrographolide or ribavirin, the supernatant of HEp-2 cells was removed, washed with PBS three times, and then incubated with 50 mU HSE for 2 h. Ultimately, RSV solution (MOI = 1) was added, and the viral load test was performed as previously described. For the detection of CX3CR1, 10 µM JMS-17-2 was added and pre-cultured with HEp-2 cells for 1 h at 37°C and 5% CO_2_. Subsequently, the supernatant fluid was discarded and washed three times with PBS. After pre-treating with andrographolide (12.5 µM) or ribavirin (6.25 µg/mL) solution for 2 h, RSV (MOI = 1) was added and incubated in a 37°C, 5% CO_2_ incubator for 24 h until the total RNA was extracted to detect the viral load (RSV F protein gene expression).

### Network pharmacology analysis

To further explore the underlying anti-RSV mechanism of andrographolide, a computer-based network pharmacological analysis approach was employed to mine the promising targets and underlying pathways related to the mediation of CX3CR1.

#### Potential targets prediction of andrographolide and RSV infections

Predictive targets of andrographolide were searched in Swiss Target Prediction (http://www.swisstargetprediction.ch/), TargetNet (https://targetnet.scbdd.com/), SuperPred (http://prediction.charite.de/), HIT-2 (http://www.badd-cao.net:2345/), Comparative Toxicogenomics Database (https://ctdbase.org/), respectively, using andrographolide as keyword. The disease perspective targets of RSV were collected in GeneCards (the Human Gene Database, https://www.genecards.org/), Online Mendelian Inheritance in Man (OMIM, https://www.omim.org/), and Therapeutic Target Database (TTD, https://db.idrblab.net/ttd/), respectively. All collected targets were standardized using the Uniprot database (https://www.uniprot.org/).

#### Common target acquisition and PPI network construction

The potential targets of andrographolide in treating RSV were intersected via Venn diagram. Then, the common targets were imported into the STRING database (https://cn.string-db.org/, version 11.5) to construct protein-protein interaction network with the setting “Homo sapiens” and “Highest confidence = 0.900. Finally, the PPI results were exported and visualized by Cytoscape software (https://cytoscape.org/, version 3.10.1). The acquirement of core targets was determined by the degree value.

### GO functions and KEGG pathway analysis

Further study was performed to investigate the underlying anti-RSV pathway of andrographolide. In short, the intersected targets were input into the Database for Annotation, Visualization, and Integrated Discovery database (https://david.ncifcrf.gov/) to conduct Gene Ontology functional including, biological process, cellular component, and molecular function, and Kyoto Encyclopedia of Genes and Genomes (KEGG) pathway analysis. The results were saved, and the visualization of the ranked high KEGG pathways (*P* value < 0.05), as well as the top 10 GO functional enrichment, was performed by an online platform (https://www.bioinformatics.com.cn/).

### Molecular docking

Before conducting molecular docking, the molecular structure of andrographolide was downloaded from the PubChem database, an open chemistry database (https://pubchem.ncbi.nlm.nih.gov/), and the structure of related targets was obtained from the RCSB Protein Data Bank (https://www.rcsb.org/). Then, CB-Dock2 (https://cadd.labshare.cn/cb-dock2/index.php, version 2, updated 2022) (71), an open online docking tool for protein-ligand blind docking, integrating cavity detection, docking, and homologous template fittings, was utilized to explore the binding affinity of andrographolide with related targets. Table S5 presented the docking information about the ligand and related receptors.

### Cellular heat migration assays

Modified CETSA was performed as previously described (72). Briefly, HEp-2 cells cultured in a cell dish were obtained. The supernatant was discarded, and the cells were washed three times with 1× PBS. Then, 1 mL of 1× PBS was added, and the cells were scraped with a cell scraper, transferred to a 1.5 mL centrifuge tube, and centrifuged for 10 min (4°C, 3,000 rpm). Then, the supernatant was discarded again, and 200 µL of lysis buffer (RIPA lysis buffer and PMSF, phosphatase inhibitor and protease inhibitor ratio of 100:1:1:1) was added to the cell pellet, mixed well, allowed to stand for 5 min, and centrifuged for 15 min (4°C, 12,000 rpm). The supernatant was collected, and its volume was recorded. The supernatants were divided into two equal parts, DMSO and andrographolide (12.5 µM) were added, respectively, and allowed to stand at room temperature for 1 h.

Afterward, each group of samples was divided into seven equal parts, with 50 µL each (45°C, 50°C, 55°C, 60°C, 65°C, 70°C, and 75°C). The samples were heated at each temperature for 8 min. After finishing heating, the samples were centrifuged for 10 min (4°C, 12,000 rpm). The supernatant was taken, and 1/4 of the extracted supernatant volume of 5× loading buffer was added. The sample was vortexed, centrifuged, and placed in a metal bath to denature at 95°C for 10 min. The sample preparation was completed. Finally, Western blotting was performed to detect the expressions of CX3CR1 proteins in the supernatant.

### Detection of inflammatory factors

To evaluate the effects of andrographolide on IL-6, IL-1β, IL-10, as well as TNF-α, before adding RSV (MOI = 1), HEp-2 cells were pretreated with andrographolide (3.125, 6.25, and 12.5 µM) or ribavirin (6.25 µg/mL), respectively, for 2 h. After 24 h, the total RNA was extracted, and the expression levels of inflammatory mediators were detected using RT-qPCR test.

### Immunofluorescence experiment

Cells were fixed using 4% paraformaldehyde (lot: 23286549, Biosharp) at room temperature for 20 min. Immunostaining was subsequently performed using a primary antibody specific to the RSV F protein (1:200). This was followed by an incubation with a fluorescent secondary antibody (1:500). Cellular nuclei were visualized by DAPI staining (0.5 µg/mL). To retain the fluorescence signals, an anti-fluorescence quencher was used as a protective layer. Samples were subsequently examined under fluorescence scanning microscope (NIB620FL, Ningbo Yongxin Optics Co., Ltd., China).

### Flow cytometry analysis

To detect the effects on ROS expression, flow cytometry analysis was performed using a BD FACSCelesta flow cytometer (Becton Dickinson and Company) equipped with 488 and 640 nm lasers. Briefly, HEp-2 cells were pretreated with andrographolide (3.125, 6.25, and 12.5 µM) or ribavirin (6.25 µg/mL) for 2 h. Subsequently, RSV solution was added and continuously cultured for 24 h at 37°C with 5% CO_2_. After 24 h, the supernatant was discarded, and the pellet was washed three times with 1× PBS. ROS fluorescent probe DCFH-DA (10 µM) was added and placed in a 37°C, 5% CO_2_ incubator in the dark for 30 min. Then, the supernatant was discarded, and the pellet was digested with EDTA for 2 min. Subsequently, 3 mL of DMEM containing 10% FBS was added, and the suspension was gently mixed by pipetting. The cells were transferred to a 10 mL sterile EP tube and centrifuged at 1,000 rpm for 5 min at room temperature. The supernatant was discarded again, and the cell pellet was washed with 1 mL of PBS. The supernatant was removed, and 500 µL of PBS was added and mixed well by pipetting. A flow cytometer was used for detection, and data analysis was performed by FlowJo software.

### Real-time reverse transcription quantitative PCR analysis

Total RNA in HEp-2 cells was extracted using Steady Pure RNA Extraction Kit, and the RNA quality was checked using Nano-100 Micro spectrophotometer. The cDNA was prepared from total RNA using the Hi Script II Q RT Super Mix for qPCR with gDNA wiper (Vazyme, Nanjing, China) according to the manufacturer’s instructions. Real-time PCR analysis was performed using the ChamQ SYBR Color Qpcr Master Mix (Vazyme, Nanjing, China) with a real-time PCR detection system (ABI Step one, Applied Biosystems) using 40 cycles of 95°C for 10 s and 60°C for 30 s. All oligonucleotide primers were synthesized by Qingke Biotech (Beijing). The sequences of primers are listed in Table 1.

**TABLE 1:** Primer sequence used in the study

Gene name	Primer sequence (5′−3′）
GAPDH	F: CTTCTTTTGCGTCGCCAGCCGA
	R: ACCAGGCGCCCAATACGACCAA
RSV-F	F: AACAGATGTAAGCAGCTCCGTTATC
	R: GATTTTTATTGGATGCTGTACATTT
RSV-G	F: CGGCAAACCACAAAGTCACA
	R: TTCTTGATCTGGCTTGTTGCA
RSV-M	F: ATGTGCTAATGTGTCCTTGGATGA
	R: TGATTTCACAGGGTGTGGTTACA
RSV-N	F: AAGGGATTTTTGCAGGATTGTTT
	R: CTCCCCACCGTAGCATTACTTG
IL-6	F: ACTCACCTCTTCAGAACGAATTG
	R: CCATCTTTGGAAGGTTCAGGTTG
IL-1β	F: CGATCACTGAACTGCACGCTC
	R: ACAAAGGACATGGAGAACACCACTT
IL-10	F: CCAAGAGAAAGGCATCTACA
	R: GGGGGTTGAGGTATCAGAG
TNF-α	F: GACGTGGAACTGGCAGAAGAG
	R: TTGGTGGTTTGTGAGTGTGAG

### Enzyme-linked immunosorbent assay

The drug effects on SOD expression were detected via commercial ELISA kits according to the requirements. Multiskan Spectrum microplate reader (Multiskan GO, Thermo Fisher Scientific [Shanghai] Instrument Co., Ltd) was used to detect the absorbance (OD) value of each well.

### Western blot

The protein expression was tested by immunoblotting according to the standard protocol. Briefly, cell lysis was achieved by treating them with RIPA buffer containing PMSF, phosphatase inhibitor, and a complete protease inhibitor at the ratios of 100:1:1. The resulting lysates were subjected to centrifugation at 12,000 rpm for 15 min at 4°C. The BCA protein assay kit was employed to determine protein concentrations in the supernatants.

After quantification, the protein samples were separated on 10% SDS-PAGE gels and then transferred onto PVDF membranes. Then, these membranes were subjected to a blocking step using a quick-sealing liquid for 20 min. Subsequently, a series of sequential incubations were carried out with the primary antibodies and their corresponding horseradish peroxidase-conjugated secondary antibodies. As a reference for loading control, GAPDH was utilized. Protein bands were visualized using enhanced chemiluminescence reagents (cat: 180-5001, Tanon) and subsequently analyzed utilizing Tanon 5200 Multi Fully Automatic Chemiluminescence Imaging System (Tanon, Shanghai, China).

### Statistical analysis

Statistical analyses were conducted using GraphPad Prism 8.0 software. Student’s *t*-tests were employed for normally distributed data to compare differences between two groups, while one-way analysis of variance with Dunnett was utilized for comparisons among multiple groups. All data are presented as the mean ± standard error from at least three independent experiments. Statistical significance was defined as follows: *P* < 0.0001, *P* < 0.001, *P* < 0.01, and *P* < 0.05.

## Data Availability

Data will be made available on request.

## SUPPLEMENTAL MATERIAL

Supplemental tables and figures spectrum.02341-25-s0001.docx Tables S1 to S5; Figures S1 and S2.

ASM does not own the copyrights to Supplemental Material that may be linked to, or accessed through, an article. The authors have granted ASM a non-exclusive, world-wide license to publish the Supplemental Material files. Please contact the corresponding author directly for reuse.
