# Supplementary material for: New insight into the epidemiological trends of respiratory syncytial virus infection and the underlying anti-respiratory syncytial virus mechanisms of andrographolide: integrating Global Burden of Disease database, network pharmacological analysis, and in vitro experiments
Source: Microbiol Spectr. 2025 Nov 13;14(1):e02341-25. doi: 10.1128/spectrum.02341-25 (PMC12772385; doi:10.1128/spectrum.02341-25)
Supplement: Supplemental tables and figures — Tables S1 to S5; Figures S1 and S2. [file spectrum.02341-25-s0001.docx]

**Supplementary Table 1.** Deaths and ASMR of RSV related LRI burdens in 1990 and 2021, as well as temporal trends during 1990 - 2021.

|  | 1990 | | 2021 | | 1990-2021 | |
| --- | --- | --- | --- | --- | --- | --- |
|  | Death number (95% UI) | Age-standardized Death rate(95% UI) | Death number  (95% UI) | Age-standardized Mortality rate(95% UI) | Cases Change | EAPC, %(95%CI) |
| Global | 139762 (123666,158111) | 2.31 (2.05,2.6) | 31525 (23348,41871) | 0.49 (0.36,0.65) | -0.77 (-0.83,-0.71) | -0.79 (-0.84,-0.73) |
| Gender |  |  |  |  |  |  |
| Male | 74232 (64778,84506) | 2.41 (2.13,2.74) | 17093 (12902,22736) | 0.52 (0.39,0.69) | -0.77 (-0.83,-0.7) | -0.78 (-0.84,-0.72) |
| Female | 65530 (57038,74889) | 2.21 (1.93,2.52) | 14432 (10608,18948) | 0.46 (0.33,0.6) | -0.78 (-0.84,-0.71) | -0.79 (-0.85,-0.73) |
| SDI rank |  |  |  |  |  |  |
| High SDI | 3391 (3102,3628) | 0.38 (0.35,0.41) | 291 (212,397) | 0.02 (0.01,0.03) | -0.91 (-0.94,-0.88) | -0.95 (-0.97,-0.93) |
| High-middle SDI | 10301 (9269,11699) | 1.16 (1.05,1.32) | 626 (467,842) | 0.06 (0.05,0.08) | -0.94 (-0.96,-0.92) | -0.95 (-0.96,-0.93) |
| Middle-SDI | 40685 (36611,45408) | 2.13 (1.93,2.37) | 3564 (2586,4722) | 0.21 (0.15,0.28) | -0.91 (-0.94,-0.88) | -0.9 (-0.93,-0.87) |
| Low-middle SDI | 49350 (42975,56450) | 2.84 (2.49,3.25) | 11773 (7764,16625) | 0.66 (0.43,0.93) | -0.76 (-0.84,-0.66) | -0.77 (-0.84,-0.67) |
| Low SDI | 35949 (29716,42706) | 3.85 (3.2,4.55) | 15265 (10767,20495) | 1.01 (0.72,1.34) | -0.58 (-0.68,-0.44) | -0.74 (-0.8,-0.66) |
| GBD regions |  |  |  |  |  |  |
| Central Asia | 4345 (4035,4708) | 4.64 (4.31,5.02) | 222 (119,365) | 0.23 (0.12,0.38) | -0.95 (-0.97,-0.92) | -0.95 (-0.97,-0.92) |
| Central Europe | 64501 (60873,67976) | 0.92 (0.87,0.96) | 9 (3,23) | 0.01 (0,0.01) | -0.99 (-1,-0.97) | -0.99 (-1,-0.98) |
| Eastern Europe | 1073 (1018,1134) | 0.68 (0.64,0.72) | 68 (30,131) | 0.04 (0.02,0.07) | -0.94 (-0.97,-0.88) | -0.94 (-0.98,-0.89) |
| High income region |  |  |  |  |  |  |
| High-income Asia Pacific | 757 (682,818) | 0.5 (0.45,0.54) | 2 (0,12) | 0 (0,0) | -1 (-1,-0.98) | -1 (-1,-1) |
| High-income North America | 968 (870,1043) | 0.3 (0.27,0.32) | 114 (56,206) | 0.02 (0.01,0.04) | -0.88 (-0.94,-0.79) | -0.93 (-0.96,-0.87) |
| Western Europe | 1292 (1159,1399) | 0.27 (0.25,0.29) | 98 (62,143) | 0.01 (0.01,0.01) | -0.92 (-0.95,-0.89) | -0.97 (-0.98,-0.95) |
| Australasia | 43 (39,46) | 0.23 (0.22,0.25) | 1 (0,1) | 0 (0,0) | -0.99 (-1,-0.97) | -1 (-1,-0.99) |
| Latin America and Caribbean |  |  |  |  |  |  |
| Andean Latin America | 1404 (1226,1590) | 2.77 (2.43,3.12) | 285 (107,465) | 0.48 (0.18,0.79) | -0.8 (-0.92,-0.65) | -0.83 (-0.93,-0.7) |

**Supplementary Table 1.** Deaths and ASMR of RSV related LRI burdens in 1990 and 2021, as well as temporal trends during 1990 - 2021 (Continued).

|  | 1990 | | 2021 | | 1990-2021 | |
| --- | --- | --- | --- | --- | --- | --- |
|  | Death number (95% UI) | Age-standardized Death rate(95% UI) | Death number  (95% UI) | Age-standardized Mortality rate(95% UI) | Cases Change | EAPC, %(95%CI) |
| Caribbean | 614 (516,733) | 1.54 (1.31,1.82) | 2 (1,12) | 0.01 (0,0.03) | -1 (-1,-0.98) | -1 (-1,-0.98) |
| Southern Latin America | 277 (263,291) | 0.59 (0.55,0.62) | 32 (14,62) | 0.04 (0.02,0.08) | -0.88 (-0.95,-0.78) | -0.93 (-0.97,-0.86) |
| Tropical Latin America | 2065 (1842,2295) | 1.37 (1.23,1.51) | 264 (78,587) | 0.12 (0.04,0.28) | -0.87 (-0.96,-0.71) | -0.91 (-0.97,-0.79) |
| Central Latin America | 2780 (2571,3032) | 1.29 (1.2,1.39) | 212 (142,305) | 0.1 (0.07,0.15) | -0.92 (-0.95,-0.89) | -0.92 (-0.95,-0.88) |
| North Africa and Middle East |  |  |  |  |  |  |
| North Africa and Middle East | 10775 (9198,13391) | 2.17 (1.85,2.67) | 1039 (560,1797) | 0.2 (0.11,0.34) | -0.9 (-0.95,-0.83) | -0.91 (-0.95,-0.84) |
| South Asia |  |  |  |  |  |  |
| South Asia | 41104 (34241,48132) | 2.09 (1.83,2.44) | 11561 (5798,18694) | 0.77 (0.39,1.25) | -0.72 (-0.86,-0.54) | -0.71 (-0.85,-0.52) |
| East, Asia, Oceania, Southeast Asia |  |  |  |  |  |  |
| East Asia | 25950 (22337,30046) | 2.34 (2.02,2.7) | 908 (513,1516) | 0.1 (0.06,0.16) | -0.96 (-0.98,-0.94) | -0.96 (-0.98,-0.93) |
| Oceania | 305 (248,380) | 3.05 (2.5,3.76) | 24 (11,44) | 0.12 (0.06,0.22) | -0.92 (-0.96,-0.86) | -0.96 (-0.98,-0.93) |
| Southeast Asia | 11749 (10245,13844) | 2.64 (2.21,3.08) | 399 (259,585) | 0.07 (0.05,0.11) | -0.97 (-0.98,-0.95) | -0.97 (-0.98,-0.95) |
| Sub-Saharan Africa |  |  |  |  |  |  |
| Central Sub-Saharan Africa | 3460 (2531,4456) | 3.31 (2.48,4.17) | 1537 (730,2437) | 0.98 (0.48,1.5) | -0.56 (-0.77,-0.31) | -0.71 (-0.85,-0.55) |
| Western Sub-Saharan Africa | 16520 (13586,19646) | 4.47 (3.68,5.3) | 9996 (5877,14969) | 1.34 (0.8,2) | -0.39 (-0.61,-0.12) | -0.7 (-0.81,-0.56) |
| Southern Sub-Saharan Africa | 1480 (1295,1694) | 2.15 (1.9,2.44) | 245 (98,490) | 0.33 (0.13,0.67) | -0.83 (-0.93,-0.67) | -0.85 (-0.94,-0.7) |
| Eastern Sub-Saharan Africa | 11960 (9744,14548) | 3.31 (2.73,3.96) | 4505 (2917,6559) | 0.85 (0.57,1.23) | -0.62 (-0.76,-0.47) | -0.74 (-0.84,-0.64) |

**Supplementary Table 2.** The number of DALYs and ASR of RSV related LRI burdens in 1990, 2021, and the temporal trend of DALYs during 1990 – 2021.

|  | 1990 | | 2021 | | 1990-2021 | |
| --- | --- | --- | --- | --- | --- | --- |
|  | DALYs number (95% UI) | Age-standardized DALY rate(95% UI) | DALYs number (95% UI) | Age-standardized DALY rate(95% UI) | Cases Change | EAPC, (95%CI) |
|  |  |  |  |  |  |  |
| Global | 12105847 (10665949,13740785) | 193.49 (170.61,219.46) | 2591507 (1902004,3468792) | 40.82 (29.91,54.59) | -0.79 (-0.84,-0.72) | -0.79(-0.84,-0.73) |
| Gender |  |  |  |  |  |  |
| Male | 6440212 (5586818,7346654) | 199.63 (173.54,227.74) | 1408575 (1048117,1888508) | 43.06 (32.05,57.79) | -0.78 (-0.84,-0.71) | -0.78 (-0.84,-0.72) |
| Female | 5665635 (4915239,6501274) | 187.13 (162.41,214.61) | 1182933 (860532,1569510) | 38.46 (27.9,51.07) | -0.79 (-0.85,-0.72) | -0.79 (-0.85,-0.73) |
| SDI rank |  |  |  |  |  |  |
| High SDI | 122762 (115755,130627) | 17.16 (16.12,18.35) | 7791 (5623,10839) | 0.89 (0.63,1.23) | -0.94 (-0.95,-0.91) | -0.95 (-0.96,-0.93) |
| High-middle SDI | 866215 (774685,990312) | 96.9 (86.68,110.78) | 28414 (21347,37667) | 3.84 (2.85,5.13) | -0.97 (-0.98,-0.96) | -0.96 (-0.97,-0.95) |
| Middle-SDI | 3563942 (3200439,3987784) | 180.15 (161.94,201.38) | 251513 (179101,340161) | 15.34 (10.88,20.86) | -0.93 (-0.95,-0.9) | -0.91 (-0.94,-0.89) |
| Low-middle SDI | 4368503 (3799151,5000857) | 244.4 (212.81,279.98) | 982202 (651938,1386106) | 52.79 (35,74.55) | -0.78 (-0.85,-0.68) | -0.78 (-0.86,-0.7) |
| Low SDI | 3177073 (2621925,3775666) | 326.85 (269.73,389.51) | 1321025 (932144,1775048) | 80.35 (56.66,107.9) | -0.58 (-0.69,-0.45) | -0.75 (-0.82,-0.68) |
| GBD regions |  |  |  |  |  |  |
| Central Asia | 386572 (358853,418956) | 410.33 (381.01,444.66) | 19064 (9879,31308) | 19.29 (10.02,31.67) | -0.95 (-0.97,-0.92) | -0.95 (-0.98,-0.92) |
| Central Europe | 64501 (60873,67976) | 73.73 (69.47,77.82) | 303 (103,666) | 0.36 (0.13,0.76) | -1 (-1,-0.99) | -1 (-1,-0.99) |
| Eastern Europe | 89677 (84820,95109) | 58.39 (55.12,61.98) | 3396 (1480,6438) | 2.55 (1.09,4.89) | -0.96 (-0.98,-0.93) | -0.96 (-0.98,-0.92) |
| High income region |  |  |  |  |  |  |
| High-income Asia Pacific | 21608 (19934,23193) | 16.94 (15.45,18.46) | 33 (3,168) | 0.01 (0,0.06) | -1 (-1,-0.99) | -1 (-1,-1) |
| High-income North America | 31180 (29641,32617) | 11.83 (11.28,12.35) | 3149 (1488,5683) | 0.96 (0.45,1.74) | -0.9 (-0.95,-0.82) | -0.92 (-0.96,-0.86) |
| Western Europe | 33033 (31405,34543) | 10.08 (9.71,10.45) | 1548 (975,2258) | 0.28 (0.18,0.41) | -0.95 (-0.97,-0.93) | -0.97 (-0.98,-0.96) |
| Australasia | 1841 (1718,1974) | 11.12 (10.34,11.99) | 11 (3,25) | 0.04 (0.01,0.09) | -0.99 (-1,-0.99) | -1 (-1,-0.99) |
| Latin America and Caribbean |  |  |  |  |  |  |

**Supplementary Table 2.** The number of DALYs and ASR of RSV related LRI burdens in 1990, 2021, and the temporal trend of DALYs during1990 - 2021 (Continued).

|  | 1990 | | 2021 | | 1990-2021 | |
| --- | --- | --- | --- | --- | --- | --- |
|  | DALYs number (95% UI) | Age-standardized DALY rate(95% UI) | DALYs number (95% UI) | Age-standardized DALY rate(95% UI) | Cases Change | EAPC, (95%CI) |
| Andean Latin America | 121705 (106006,138173) | 225.47 (196.65,255.68) | 16774 (6358,28740) | 27.78 (10.53,47.55) | -0.86 (-0.95,-0.75) | -0.88 (-0.95,-0.78) |
| Caribbean | 51754 (43056,62437) | 123 (102.62,147.91) | 161 (47,809) | 0.41 (0.12,2.06) | -1 (-1,-0.98) | -1 (-1,-0.98) |
| Southern Latin America | 19127 (18028,20171) | 38.14 (35.97,40.18) | 920 (398,1779) | 1.67 (0.74,3.26) | -0.95 (-0.98,-0.91) | -0.96 (-0.98,-0.92) |
| Tropical Latin America | 175597 (156002,196127) | 109.81 (97.68,122.46) | 12371 (3600,27804) | 6.58 (1.93,14.88) | -0.93 (-0.98,-0.84) | -0.94 (-0.98,-0.86) |
| Central Latin America | 240956 (222737,263719) | 104.04 (96.2,113.67) | 15564 (10262,22530) | 7.93 (5.23,11.48) | -0.94 (-0.96,-0.9) | -0.92 (-0.95,-0.89) |
| North Africa and Middle East |  |  |  |  |  |  |
| North Africa and Middle East | 953149 (813178,1186438) | 185.69 (158.49,230.93) | 79031 (40379,138868) | 13.74 (7.08,24.08) | -0.92 (-0.96,-0.85) | -0.93 (-0.96,-0.86) |
| South Asia |  |  |  |  |  |  |
| South Asia | 3645872 (3031824,4273298) | 228.41 (190.32,267.49) | 959424 (484295,1553206) | 4.89 (3.2,7.28) | -0.74 (-0.87,-0.57) | -0.72 (-0.86,-0.55) |
| East, Asia, Oceania, Southeast Asia |  |  |  |  |  |  |
| East Asia | 2268332 (1947323,2627319) | 198.61 (170.68,229.97) | 45581 (25631,76212) | 6.68 (3.69,11.12) | -0.98 (-0.99,-0.97) | -0.97 (-0.98,-0.94) |
| Oceania | 27063 (21929,33795) | 259.24 (210.3,322.38) | 2105 (1011,3885) | 10.51 (5.05,19.34) | -0.92 (-0.96,-0.87) | -0.96 (-0.98,-0.93) |
| Southeast Asia | 1030724 (898445,1218338) | 177.34 (154.64,209.25) | 27229 (17729,40561) | 63 (31.79,102.03) | -0.97 (-0.98,-0.96) | -0.97 (-0.98,-0.96) |
| Sub-Saharan Africa |  |  |  |  |  |  |
| Central Sub-Saharan Africa | 305072 (222276,393720) | 272.16 (199.13,350.26) | 128480 (59859,204950) | 65.72 (31.33,103.94) | -0.58 (-0.79,-0.35) | -0.76 (-0.88,-0.63) |
| Western Sub-Saharan Africa | 1457578 (1198456,1733011) | 379.38 (309.14,452.76) | 872819 (513361,1305846) | 108.55 (63.85,162.6) | -0.4 (-0.62,-0.13) | -0.71 (-0.82,-0.58) |
| Southern Sub-Saharan Africa | 127067 (110619,146146) | 172.02 (150.42,197.12) | 19442 (7746,39010) | 24.96 (9.96,50.12) | -0.85 (-0.94,-0.7) | -0.85 (-0.94,-0.71) |
| Eastern Sub-Saharan Africa | 1053436 (856319,1285137) | 272.46 (221.83,331.61) | 384102 (246486,559267) | 62.48 (40.51,90.9) | -0.64 (-0.77,-0.48) | -0.77 (-0.85,-0.68) |

**Supplementary Table 3.** AAPC of ASMR for RSV related LRI disease burden from 1990 to 2021.

|  | Gender | Period | ASMR AAPC (95% CI) |
| --- | --- | --- | --- |
| Global | Both | 1990-2021 | -5.26 (-5.49,-5.02)* |
|  | Female |  | -5.37 (-5.62,-5.12)* |
|  | Male |  | -5.12 (-5.33,-4.91)* |
| High SDI | Both | 1990-2021 | -6.87 (-7.72,-6.02)* |
|  | Female |  | -6.87 (-7.72,-6.02)* |
|  | Male |  | -7.15 (-8.09,-6.21)* |
| High-middle SDI | Both | 1990-2021 | -8.47 (-8.81,-8.12)* |
|  | Female |  | -8.77 (-9.05,-8.49)* |
|  | Male |  | -8.10 (-8.47,-7.72)* |
| Middle-SDI | Both | 1990-2021 | -7.27 (-7.61,-6.94)* |
|  | Female |  | -7.39 (-7.66,-7.12)* |
|  | Male |  | -7.15 (-7.40,-6.91)* |
| Low-middle SDI | Both | 1990-2021 | -5.35 (-5.63,-5.06)* |
|  | Female |  | -5.48 (-5.77,-5.20)* |
|  | Male |  | -5.21 (-5.49,-4.93)* |
| Low SDI | Both | 1990-2021 | -4.55 (-4.95,-4.14)* |
|  | Female |  | -4.81 (-5.19,-4.43)* |
|  | Male |  | -4.28 (-4.44,-4.11)* |

APC with * indicates statistical significance (*P* < 0.05).

**Supplementary Table 4.** Detection results of TCID50.

| Dilution of RS virus solution | Wells with CPE | Wells without CPE | Cumulative wells with CPE | Cumulative wells without CPE | Rates of CPE (%) |
| --- | --- | --- | --- | --- | --- |
| 10^-1^ | 6 | 0 | 24 | 0 | 100 |
| 10^-2^ | 6 | 0 | 18 | 0 | 100 |
| 10^-3^ | 6 | 0 | 12 | 0 | 100 |
| 10^-4^ | 5 | 1 | 6 | 1 | 85.7 |
| 10^-5^ | 1 | 5 | 1 | 6 | 16.7 |
| 10^-6^ | 0 | 6 | 0 | 12 | 0 |
| 10^-7^ | 0 | 6 | 0 | 18 | 0 |
| 10^-8^ | 0 | 6 | 0 | 24 | 0 |

**Supplementary Table 5.** Docking information about the ligand and receptor.

| **Ligand** | **Receptor** | **Cavity Volume (Å^3^)** | **Center (x, y, z)** | **Docking size (x, y, z)** | **Vina score** |
| --- | --- | --- | --- | --- | --- |
| Andrographolide  (PubChem CID: 5318517) | F protein (7UJ3) | 1136 | 182.548, 170.758, 187.972 | 22, 22, 22 | -7.1 |
|  | G protein (5WN9) | 410 | -10.47, -19.915, 16.175 | 22, 22, 22 | -7.4 |
|  | M protein (4D4T) | 115 | 26.223, 18.729, -2.474 | 22, 22, 22 | -6.2 |
|  | N protein (8OP1) | 4824 | 247.555, 208.872, 264.931 | 35, 22, 35 | -8.1 |
|  | CX3CR1 (7XBW) | 2126 | 103.437, 158.351, 128.422 | 31, 22, 28 | -9.1 |
|  | CD44 (1UUH) | 648 | -5.686, -13.251, -0.184 | 22, 22, 22 | -7.0 |
|  | HPB (1AE5) | 148 | 4.371, 22.877, 40.785 | 22, 22, 22 | -7.4 |
|  | TNF (5UUI) | 216 | 48.19, 64.531, 12.837 | 22, 22, 22 | -6.3 |
|  | IL-6 (1ALU) | 722 | 5.783, -24.221, 17.667 | 22, 22, 22 | -6.0 |
|  | STAT3 (6NJS) | 525 | 2.262, 25.567, 29.118 | 22, 22, 22 | -7.4 |
|  | IL-1β (5R7W) | 189 | 39.941, 8.943, 56.81 | 22, 22, 22 | -6.5 |
|  | TP 53 (6MY0) | 4009 | 18.771, 20.654, 60.817 | 29, 35, 22 | -6.1 |
|  | AKT 1 (5WBL) | 887 | -20.281, -20.281, 20.587 | 22, 22, 29 | -8.3 |
|  | NF-κB1 (8TQD) | 792 | 14.373, -7.283, -5.437 | 22, 22, 22 | -6.3 |
|  | JUN (2P33) | 2884 | 22.659, 16.042, 29.161 | 22, 30, 22 | -8.8 |
|  | BCL-2 (5WHH) | 142 | -10.364, 42.327, 29.192 | 22, 22, 22 | -6.9 |
|  | IFNG (5SEG) | 1768 | -48.145, 50.319, 10.168 | 22, 22, 29 | -8.6 |
|  | IL-10 (2ILK) | 808 | 6.629, 53.478, 35.397 | 22, 22, 22 | -7.1 |


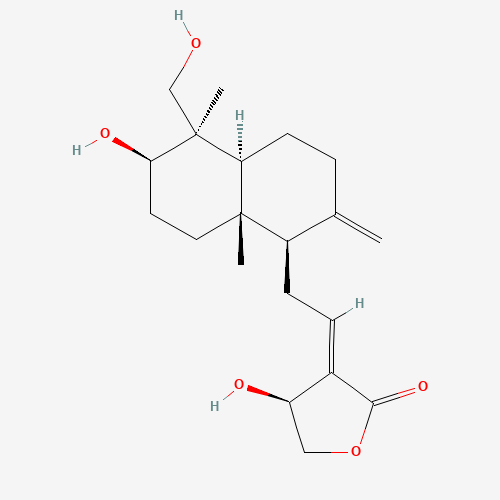


**Supplementary Figure 1.** The molecular structure of andrographolide (PubChem CID: 5318517, Molecular formula: C_20_H_30_O_5_, CAS number: 5508-58-7).


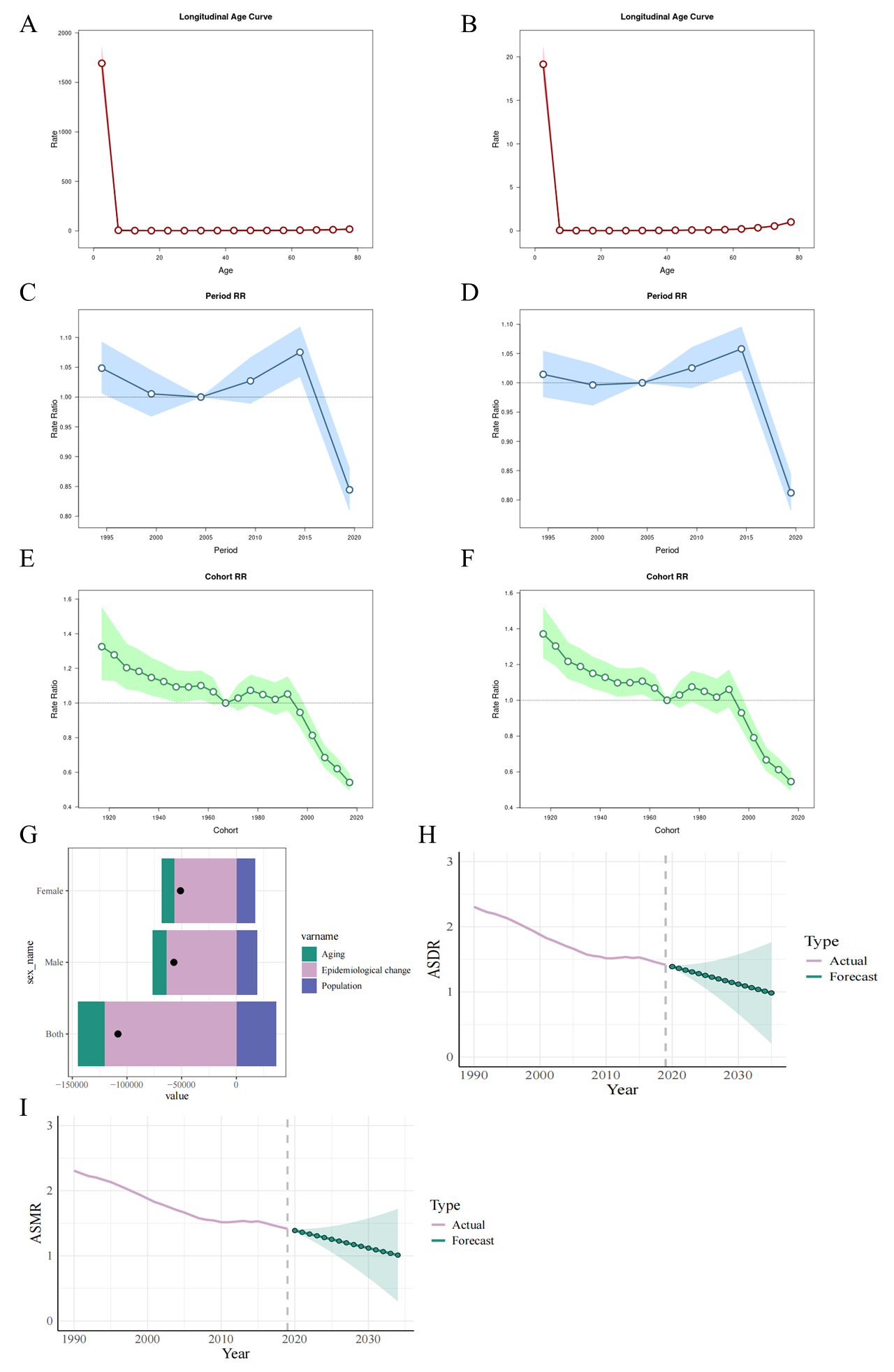


**Supplementary Figure 2.** Age period cohort model analysis of global RSV infection mortality and DALY rates from 1992 to 2021, and prediction of disease burden of LRI caused by RSV worldwide from 2020 to 2034. (A) Period effect of mortality rate. (B) Age effect of mortality rate. (C) DALYs rate age effect. (D) Mortality queue effect. (E) DALYs rate queue effect. (F) Period effect of DALYs rate. (G) Decomposition analysis of different genders, ages, and trends. (H) Predicting the disease burden of LRI caused by RSV worldwide from 2020 to 2034 based on ASDR. (I) Predicting the disease burden of LRI caused by RSV worldwide from 2020 to 2034 based on ASMR.
